# ACADM-mediated fatty acid β-oxidation pathway in atherosclerosis and abdominal aortic aneurysm

**DOI:** 10.1093/eurheartj/ehag372

**Published:** 2026-06-05

**Authors:** Hengyuan Zhang, Lingyue Sun, Tingfeng Xu, Xiaowen Hou, Fangyuan Zhong, Fanchao Shi, Yifan Chen, Yinan Li, Yuli Wang, Yuyan Lyu, Wei Zhang, Yuhua Fu, Yichao Zhao, Ancai Yuan, Lei Chen, Yong Ji, Minxian Wang, Boxun Lu, Jun Pu

**Affiliations:** Department of Cardiology, Renji Hospital, School of Medicine, State Key Laboratory of Systems Medicine for Cancer, Shanghai Cancer Institute, Shanghai Jiao Tong University, 160 Pujian Road, Shanghai200127, China; Department of Cardiology, Renji Hospital, School of Medicine, State Key Laboratory of Systems Medicine for Cancer, Shanghai Cancer Institute, Shanghai Jiao Tong University, 160 Pujian Road, Shanghai200127, China; China National Center for Bioinformation, Beijing Institute of Genomics, Chinese Academy of Sciences, University of Chinese Academy of Sciences, Beijing, China; Department of Cardiology, Renji Hospital, School of Medicine, State Key Laboratory of Systems Medicine for Cancer, Shanghai Cancer Institute, Shanghai Jiao Tong University, 160 Pujian Road, Shanghai200127, China; Central Laboratory, Ningbo Hangzhou Bay Hospital (Ningbo Branch of Renji Hospital, Shanghai Jiao Tong University School of Medicine, Shanghai, Ningbo, China; Department of Cardiology, Renji Hospital, School of Medicine, State Key Laboratory of Systems Medicine for Cancer, Shanghai Cancer Institute, Shanghai Jiao Tong University, 160 Pujian Road, Shanghai200127, China; Department of Cardiology, Renji Hospital, School of Medicine, State Key Laboratory of Systems Medicine for Cancer, Shanghai Cancer Institute, Shanghai Jiao Tong University, 160 Pujian Road, Shanghai200127, China; Department of Cardiology, Renji Hospital, School of Medicine, State Key Laboratory of Systems Medicine for Cancer, Shanghai Cancer Institute, Shanghai Jiao Tong University, 160 Pujian Road, Shanghai200127, China; Department of Vascular Surgery, Renji Hospital, School of Medicine, Shanghai Jiao Tong University, Shanghai, China; Department of Vascular Surgery, Renji Hospital, School of Medicine, Shanghai Jiao Tong University, Shanghai, China; Department of Cardiology, Renji Hospital, School of Medicine, State Key Laboratory of Systems Medicine for Cancer, Shanghai Cancer Institute, Shanghai Jiao Tong University, 160 Pujian Road, Shanghai200127, China; Department of Cardiology, Renji Hospital, School of Medicine, State Key Laboratory of Systems Medicine for Cancer, Shanghai Cancer Institute, Shanghai Jiao Tong University, 160 Pujian Road, Shanghai200127, China; State Key Laboratory of Medical Neurobiology and MOE Frontiers Center for Brain Science, School of Life Sciences, Fudan University, Shanghai, China; Department of Cardiology, Renji Hospital, School of Medicine, State Key Laboratory of Systems Medicine for Cancer, Shanghai Cancer Institute, Shanghai Jiao Tong University, 160 Pujian Road, Shanghai200127, China; Department of Cardiology, Renji Hospital, School of Medicine, State Key Laboratory of Systems Medicine for Cancer, Shanghai Cancer Institute, Shanghai Jiao Tong University, 160 Pujian Road, Shanghai200127, China; Department of Cardiology, Renji Hospital, School of Medicine, State Key Laboratory of Systems Medicine for Cancer, Shanghai Cancer Institute, Shanghai Jiao Tong University, 160 Pujian Road, Shanghai200127, China; Key Laboratory of Drug Targets and Translational Medicine for Cardio-cerebrovascular Diseases, Nanjing Medical University, Nanjing, China; State Key Laboratory of Frigid Zone Cardiovascular Diseases (SKLFZCD), Harbin Medical University, Harbin, China; China National Center for Bioinformation, Beijing Institute of Genomics, Chinese Academy of Sciences, University of Chinese Academy of Sciences, Beijing, China; State Key Laboratory of Medical Neurobiology and MOE Frontiers Center for Brain Science, School of Life Sciences, Fudan University, Shanghai, China; Department of Cardiology, Renji Hospital, School of Medicine, State Key Laboratory of Systems Medicine for Cancer, Shanghai Cancer Institute, Shanghai Jiao Tong University, 160 Pujian Road, Shanghai200127, China

**Keywords:** Atherosclerosis, Abdominal aortic aneurysm, Lipid metabolism, Machine learning, Lipid droplet

## Abstract

**Background and Aims:**

Atherosclerosis (AS) and abdominal aortic aneurysm (AAA) are both metabolism-associated vascular diseases, yet the role of lipid metabolic disturbances in their pathogenesis remains largely unknown. This study aimed to clarify the differential impact of lipid metabolic disturbances and their underlying mechanisms in AS and AAA.

**Methods:**

Lipidomic analysis was performed to identify lipid metabolic differences between AS and AAA in various mouse models and different human cohorts. A multi-omics approach, integrated with functional assays, was utilized to elucidate the downstream mechanisms underlying disease-specific lipid metabolic features. Machine learning models were developed based on lipidomic features to differentiate AS from AAA.

**Results:**

Lipidomic analysis of mouse models and human samples revealed a predominant enrichment of neutral lipids (e.g. triglycerides and cholesterol esters) in AS, in contrast to phosphoglycerides in AAA. Consistently, large-scale longitudinal data from the UK Biobank showed strong positive associations of triglycerides, cholesterol, and fatty acid with the future risk of coronary atherosclerotic disease, while phosphoglycerides were negatively associated with the risk of AAA. Integrated transcriptomic and metabolomic analyses identified fatty acid metabolism, particularly *Acadm*-mediated fatty acid β-oxidation (FAO) pathway, as the most significantly altered lipid metabolic pathway contributing to the lipid metabolic differences between AS and AAA. Consistently, targeted restoration of the *Acadm*-mediated FAO pathway inhibited AS by reducing lipotoxic metabolites and preserving mitochondrial homeostasis but had little impact on AAA. Further validation with lipid droplets autophagy-tethering compound (LD·ATTEC), which selectively eliminates intracellular lipid droplets, significantly alleviated AS progression and improved FAO with no significant change observed in AAA. Finally, predictive models were developed based on lipidomic features using machine learning algorithms, facilitating accurate differentiation between these two vascular diseases.

**Conclusions:**

These findings define previously unrecognized distinct lipid metabolic characteristics in the pathogenesis of AS vs AAA, providing a basis for differential diagnosis and targeted treatments.

## Structured Graphical Abstract

**Structured Graphical Abstract:**
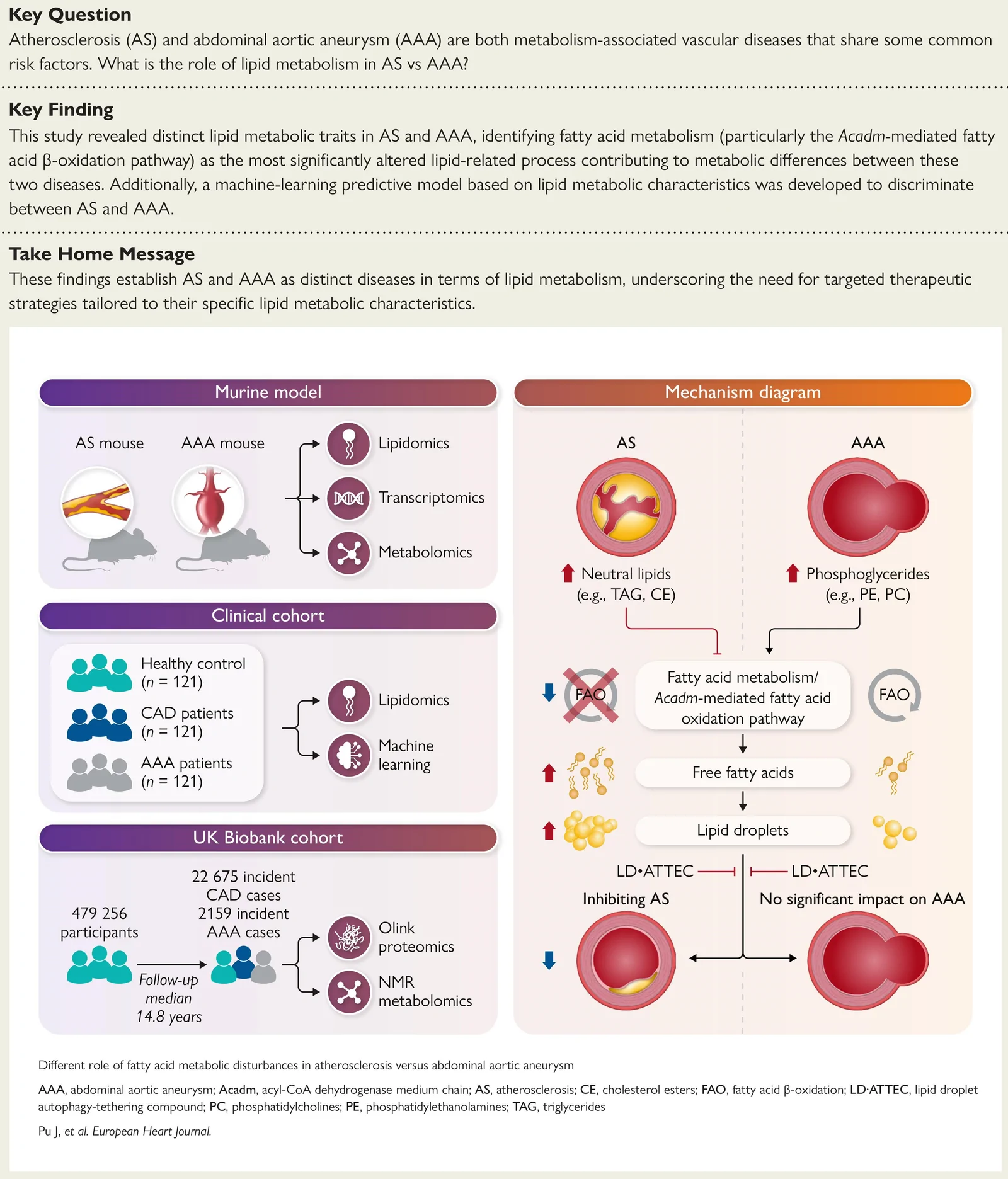

Translational perspective This study demonstrated that atherosclerosis (AS) and abdominal aortic aneurysm (AAA) were distinct vascular diseases in terms of lipidomic characteristics in both mouse models and human cohorts. Mechanistic studies identified *Acadm*-mediated fatty acid β-oxidation as the key disease-specific pathway contributing to these differences between AS and AAA. The findings that *Acadm* protected against AS in animal models were further supported by the human whole-exome sequencing data from the UK Biobank cohort, which demonstrated that loss-of-function mutations in *ACADM* were associated with increased AS burden. Furthermore, machine learning models based on their respective lipidomic features effectively discriminated between AS and AAA, highlighting the translational potential of lipid metabolism-based classifiers for precision diagnosis and targeted treatment strategies.

## Introduction

Atherosclerosis (AS) and abdominal aortic aneurysm (AAA) are both severe vascular pathologies characterized by structural and functional alterations of the arterial wall, predisposing to impaired blood flow and life-threatening complications. Atherosclerosis and AAA often coexist and share several major risk factors (e.g. advanced age, male sex, smoking, and hypertension), suggesting a potential link between the two conditions. However, accumulating evidence has suggested that AS and AAA should be distinguished, exhibiting distinct pathophysiological responses to genetic predisposition, biomechanical factors, and inflammatory response.^1–4^ For instance, AS is initiated by endothelial activation, followed by lipid accumulation, inflammation and calcification, ultimately leading to luminal narrowing.^5^ Conversely, AAA is characterized by vascular smooth muscle cell dysfunction in the medial, accompanied by elastin fragmentation and extracellular matrix remodelling.^6^ Moreover, human genome-wide sequencing has revealed distinct pathogenic loci in AS and AAA,^1^ and previous animal studies showed several inflammatory cytokines exert opposite effects in the two diseases.^2^

Importantly, although AS and AAA are both considered as metabolism-associated vascular diseases, the differential impact of lipid metabolic disturbances on AS and AAA remains largely unknown. For example, dyslipidaemia is a well-established risk factor of AS, but its role in AAA remains controversial with studies reporting both positive and negative associations.^7–9^ Furthermore, clinical trials investigating the efficacy of lipid-lowering interventions with statins in slowing AAA growth have produced inconsistent results.^10,11^ Although current practice recommends statin use in AAA patients to manage concomitant cardiovascular risk,^12^ some studies suggest that lipid-lowering strategies may not alter AAA progression or even associated higher incidence of AAA.^8,13^ As a result, medications effective in managing AS cannot be assumed to limit AAA progression.^14^ Clarifying the distinct lipid metabolic characteristics and their underlying mechanisms in AS and AAA is urgently needed to develop more specific and effective treatments.

Thus, while previous studies have shown differences in the overall pathomechanisms of AS and AAA, the precise role of lipid metabolism in these differences remains unclear. This study aims to address this gap by systematically characterizing disease-specific lipidomic differences in AS and AAA in both animal models and human cohorts and identify the underlying mechanisms. Based on the specific lipidomic features, we sought to develop machine learning models facilitating accurate diagnosis and differential diagnosis between these two vascular diseases.

## Methods

### Animal studies

All animal experiments were conducted in accordance with the National Institutes of Health Guidelines on the Care and Use of Laboratory Animals (NIH Publication, 8th Edition, 2011) and were approved by the Animal Ethics Committee of Renji Hospital (RJ2022-1211). Both male and female mice aged 8–12 weeks were used in this study. *ApoE*^−/−^ mice on a C57BL/6J background were kept in a specific pathogen-free facility under controlled setting (temperature: 22 ± 2°C; relative humidity: 55 ± 15%; light/dark cycle: 12/12 h) and provided *ad libitum* access to food and water. Two AS animal models, induced by wire injury- or high-fat diet (HFD, 40% kcal fat and 1.25% cholesterol), and two AAA animal models, induced by angiotensin II (Ang II) or calcium phosphate (CaPO_4_), were established. For all experiments, animals were randomly assigned to experimental groups using simple randomization, ensuring age and sex matching.

Ang II- or CaPO_4_-induced AAA models were established as described in our previous studies.^15,16^ To generate an Ang II-induced AAA model, *ApoE*^−/−^ mice were infused with Ang II (1000 ng/kg/min; A9525; Sigma, USA) via subcutaneous osmotic minipumps while being maintained on an HFD for 4 weeks. To generate a CaPO_4_-induced AAA model, the infrarenal region of the abdominal aorta of the *ApoE^−/−^* mice was treated with 0.5 mol/L calcium chloride for 10 min, followed by phosphate-buffered saline for another 5 min to form CaPO_4_. An aneurysm was defined as a diameter increase of at least 50%. To compare with AAA, an abdominal aortic AS mouse model was generated using a wire injury plus 4-week HFD approach to induce atherosclerotic plaque formation in the abdominal aorta.^17^ Briefly, a 0.36-mm guide wire was advanced into the abdominal aorta under ultrasound guidance and then retracted and advanced in a sawing motion 20–30 times to injure the endothelium of the abdominal aorta, followed by an HFD for 4 weeks. To confirm findings from wire injury-induced AS model, a diet-induced AS mouse model was established by maintaining *ApoE^−/−^* mice on an HFD for 12 weeks.^18^

### Human studies

All human samples used in this study were processed under Institutional Review Board approved protocols at Shanghai Jiao Tong University. All study participants were recruited after providing informed consent with approval by the Ethics Committee of Renji Hospital, School of Medicine, Shanghai Jiao Tong University (KY2021-129-B, KY2019-101), and the study was conducted according to the criteria set by the Declaration of Helsinki (2013).

#### Renji Hospital study

A total of 363 study participants from Renji Hospital, Shanghai Jiao Tong University, were enrolled in this study between December 2018 and December 2022. The inclusion criteria for coronary artery disease (CAD) cases were (i) coronary artery stenosis of ≥50% confirmed by coronary computed tomography angiography (CTA) and (ii) exclusion of any aortic diseases, such as aortic aneurysm and aortic dissection, as confirmed by CTA. The inclusion criteria for AAA cases were (i) abdominal aortic diameters of ≥30 mm measured by CTA, in accordance with the Society for Vascular Surgery Practice Guidelines,^19^ and (ii) exclusion of any atherosclerotic diseases, including CAD, stroke, and peripheral artery diseases, as confirmed by CTA. To eliminate potential confounding effects of lipid-lowering medications, only patients with a first-time diagnosis of CAD or AAA were included, and those with a history of any atherosclerotic diseases and aortic diseases and using lipid-modifying drugs were excluded from the study. During the same period, control subjects were identified from individuals undergoing routine health examinations with CTA screening or those with suspected cardiovascular disease but were finally excluded after CTA examinations. Characteristics collected for each participant and the clinical characteristics of the study subjects are detailed in Supplementary data online, *Table S1*. Plasma samples from all participants were obtained after overnight fasting and stored at −80°C until further analysis.

#### UK Biobank studies

To further investigate the longitudinal associations of lipid traits with the incidence of CAD and AAA, we used the data from the UK Biobank. The UK Biobank is a prospective, population-based cohort study that collected health-related data from 502 241 participants aged 40–69 years at baseline (2006–10).^20^ Additionally, plasma samples for metabolomics and proteomics were collected at baseline. The current research has been conducted using the UK Biobank Resource under the application ID 89885. Incident CAD and AAA cases were collected through any primary care data, hospital admission records, and death register records. In our study, hospital inpatient data were coded according to the International Classification of Diseases, Ninth/Tenth Version codes. All operations and procedures were coded according to the Office of Population, Censuses and Surveys: Classification of Interventions and Procedures (OPCS-4) codes. The specific information is shown in Supplementary data online, *Table S2*. Given that the UK Biobank includes both baseline and follow-up data, we focused on longitudinal analyses to assess the associations between baseline metabolite levels and the risks of CAD and AAA onset. The follow-up period was defined from the date of recruitment, which served as the origin time for survival analyses, to the date of CAD or AAA diagnosis, death, loss of follow-up, or end of follow-up (1 January 2024), whichever came first. Participants who remained event-free were censored at death, loss to follow-up, or the administrative end of follow-up.

### Statistical analysis

Orthogonal projections to latent structures-discriminant analysis (OPLS-DA) were conducted using SIMCA software (version14.0, Sartorius Stedim Data Analytics AB, Sweden). Model validity was evaluated using seven-fold cross-validation based on R^2^Y and Q^2^ values, and permutation testing (200 iterations) was performed to assess robustness and exclude overfitting. Significantly regulated metabolites and lipids were identified by variable importance in projection (VIP) scores > 1 and *P*-value < .05 from two-group comparisons using Student’s *t*-test, Welch’s *t*-test, or Mann–Whitney *U* test according to data distribution and variance assumptions, as described below. Descriptive statistics were employed to summarize clinical and demographic characteristics. Continuous variables were expressed as mean ± standard deviation (SD) for approximately symmetric distributions and as median (interquartile range, IQR) for skewed distributions. Categorical variables were presented as frequency (percentage). Effect estimates are reported with corresponding 95% confidence intervals (CIs). The normality of data distribution was assessed using Q-Q plots and the Shapiro–Wilk test. For two-group comparisons, homogeneity of variances was evaluated using Levene’s test. Normally distributed data were analysed using Student’s *t*-test (for equal variances) or Welch’s *t*-test (for unequal variances), whereas non-normally distributed data were analysed using Mann–Whitney *U* test. For comparisons among three or more groups, homogeneity of variances was assessed using the Brown–Forsythe test. Normally distributed data were analysed using one-way ANOVA with Tukey’s *post hoc* test (for equal variances) or Welch’s ANOVA with Tamhane’s T2 *post hoc* test (for unequal variances). Two-way ANOVA with Tukey’s *post hoc* analysis was used when more than two groups and variables were compared. For non-normally distributed data, the Kruskal–Wallis test was applied. Correlations were assessed using both Spearman’s rank correlation (ρ) and Pearson correlation (*r*). Linear regression lines were fitted using ordinary least squares (OLS) for visualization purposes only. Bivariate comparisons of categorical variables were performed with the *χ*² test (all expected cell counts were ≥5) or with the Fisher’s exact test (any expected cell count was <5). Associations between treatment (vehicle vs LD·ATTEC) and systolic blood pressure over time in AS and AAA mice were assessed using linear generalized estimating equations. Representative images were chosen to represent the average results achieved within the experiment. All statistical analyses were performed using GraphPad Prism (version 8), Python (3.10.14), or R (version 4.3.2), with a *P*-value < .05 considered statistically significant.

For UK Biobank data, hazard ratios and 95% CIs were calculated using Cox proportional hazards models with the date of recruitment as the origin time, adjusted for gender, age at enrolment, body mass index (BMI), smoking status, medication use, and assessment centre. Covariate selection was guided by a directed acyclic graph (DAG) constructed based on established cardiovascular risk determinants summarized in the PREVENT study.^21^ For the proteomics analysis, we also accounted for the first 10 genetic principal components and genotype arrays. The proportional hazards assumption was evaluated using Schoenfeld’s tests, and linearity of quantitative predictors was assessed using restricted cubic splines. To ensure consistency in baseline characteristics across CAD, AAA, and control groups, propensity scores were estimated using a multivariable logistic regression model and used for matching to generate matched cohorts. Age, sex, BMI, current smoking status, and medication use were included as variables in the propensity score model to estimate scores for matching. Matching was then performed using 1:3 nearest-neighbour matching with a caliper width equal to 0.2 of the SD of the logit of the propensity score.^22,23^ For the matched cohorts, associations were estimated using Cox proportional hazards models incorporating a shared frailty term at the matched-set level. Since AAA group had the least number of participants, we randomly selected three matched CAD participants and three matched controls for each AAA participant. Absolute standardized mean differences were calculated to compare differences between matched groups, with >10% suggesting imbalance. Analysis was further adjusted for competing risks using the Fine and Gray proportional sub-distribution hazards regression model, with incident CAD and all-cause death specified as competing events in the AAA analysis, and incident AAA and all-cause death in the CAD analysis. The proportions of competing events were 11.47% and 7.23%, respectively. Differences in association magnitude for incident CAD and AAA were estimated using Z_diff_ = (β_CAD_-β_AAA_)/$\sqrt{\left(\mathrm{se}2\mathrm{CAD}+\mathrm{se}2\mathrm{AAA}\right)}$.^24^ A control group sample-splitting sensitivity analysis was additionally performed to avoid covariance arising from the shared controls. Sample size sufficiency was evaluated using the FDRsamplesize2 method for both lipidomic analysis and Cox proportional hazards analysis.^25^

## Results

### Lipid metabolism differs between atherosclerosis and abdominal aortic aneurysm mouse models

The divergent role of lipid metabolic disturbances in the pathogenesis of AS vs AAA remains largely unknown. To elucidate this, we first established two mouse models of AS: one induced by wire injury surgery with a 4-week HFD^17^ and the other by continuous HFD for 12 weeks.^26^ Atherosclerosis severity was assessed by measuring the percentage of aortic lesion area. We analysed correlations between plasma lipid levels (including total cholesterol, triglycerides, and LDL cholesterol) and AS severity and found a strong positive correlation between elevated these plasma lipids and AS progression in both models (see Supplementary data online, *Figure S1A* and *B*). We also developed two AAA models: the Ang II-infused model^15,16^ and the CaPO_4_-induced model.^15,16^ Abdominal aortic aneurysm severity was measured by the maximal diameter of the suprarenal abdominal aorta. In contrast to AS, we observed weak correlations between lipid levels and AAA severity in both models (see Supplementary data online, *Figure S1C* and *D*). These results suggest that lipid levels have a stronger correlation with AS than with AAA in mouse models. Given the differential involvement of lipid metabolism in AS and AAA, we performed lipidomic analyses on mouse aortic tissues to define disease-specific lipid metabolic features. Lipidomic is a high-throughput analytical technique widely used to study lipid metabolism by quantifying changes in specific lipid classes and their concentrations.^27^ For comparison, we selected the wire injury-induced abdominal aortic AS model and the Ang II-induced AAA model (*Figure 1A* and *B*), as both target the same lesion site in the abdominal aorta and follow the same dietary intervention. The lipidomic analysis identified 328 significantly altered lipid metabolites in AS and 115 in AAA (*Figure 1C*). Analysis of the number of altered lipid metabolites revealed that the top lipid class in AS were glycerolipids (with 186 altered lipid metabolites), accounted for 56.7% of the total altered lipid metabolites. In contrast, in AAA, phosphoglycerides were the top lipid class (with 63 altered lipid metabolites), constituting 54.7% of the total altered lipid metabolites (*Figure 1D*). Of the 23 lipid subclasses detected, 15 displayed significant changes in AS and 16 in AAA. Triglycerides were the most significantly elevated lipid subclass in AS, followed by cholesterol esters, while phosphatidylethanolamines were the most prominently increased lipid subclass in AAA, followed by phosphatidylcholines (*Figure 1E–H*). Direct comparison between AS and AAA identified 40 significantly differentially expressed lipid metabolites (*Figure 1I*; Supplementary data online, *Figure S2*), belonging to three lipid classes (glycerolipids, fatty acyls, and sterol lipids) (*Figure 1J*). Further lipid subclass analysis revealed that triglycerides, diglycerides, free fatty acids, and cholesterol esters exhibited significant increase in AS (vs AAA) (*Figure 1K* and *L*). Together, our lipidomic data demonstrated a pronounced increase of neutral lipids (e.g. triglycerides and cholesterol esters) in AS, while phosphoglycerides (e.g. phosphatidylethanolamines and phosphatidylcholines) were more prominently elevated in AAA.

**Figure 1:**
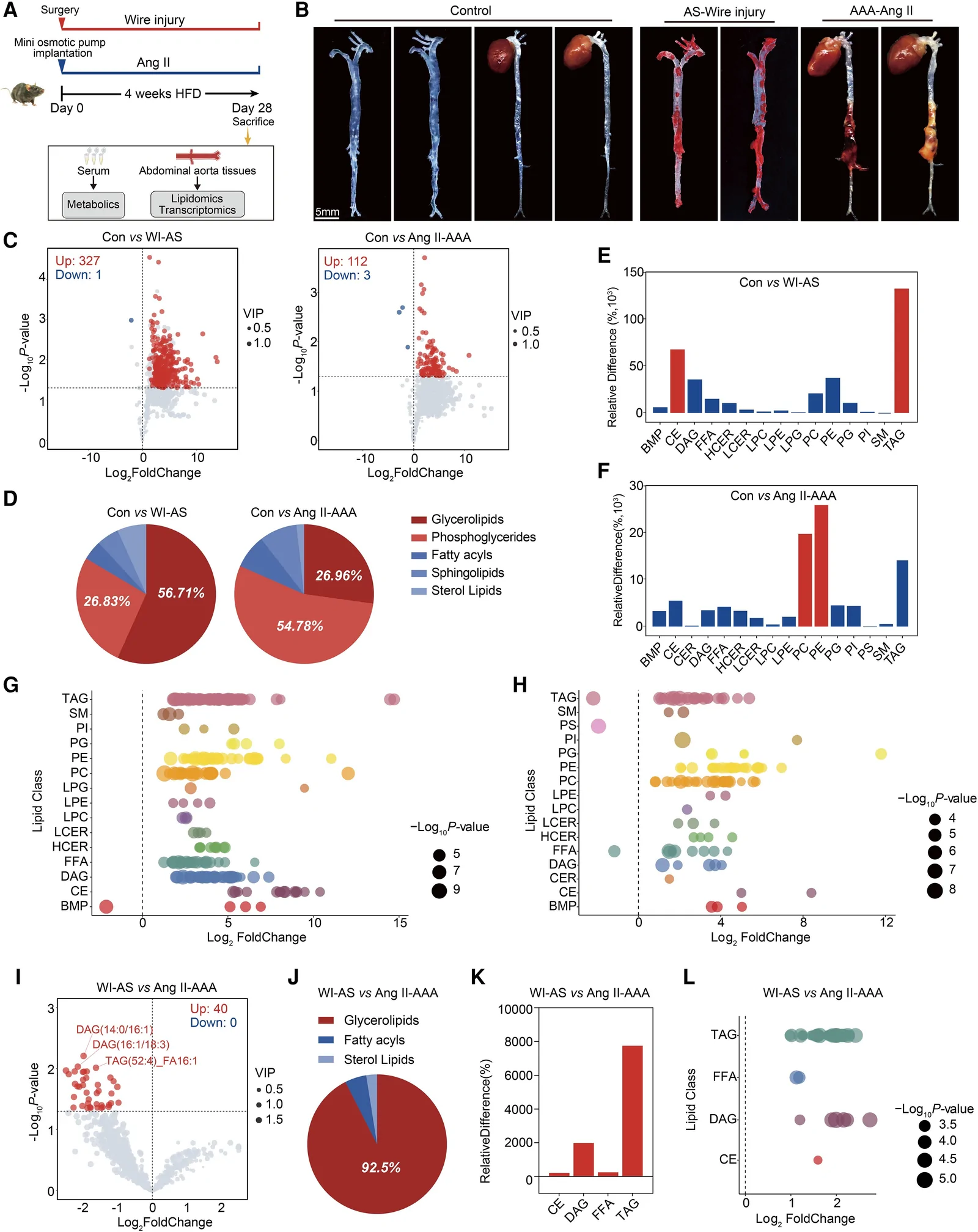
Lipid metabolism differs between AS and AAA mouse models. (*A*) Schematic illustrations of the animal experimental design. (*B*) Representative images of macroscopic features and ORO staining of abdominal aortas. (*C*) Volcano plots showing the lipidomic alterations in AS and AAA mice compared to controls (*n* = 5 mice/group). Coloured areas highlight lipids with a *P <* .05 and VIP > 1. (*D*) Pie charts showing the composition of the altered lipid metabolites by lipid classes in AS and AAA mice compared to controls. (*E* and *F*) Bar plots showing the degree of lipid metabolite changes in AS and AAA mice compared to controls. Red bars represent the top two most significantly altered lipid subclasses. (*G* and *H*) Dot plots showing log_2_ fold changes in lipid subclasses in AS and AAA mice compared to controls, and the corresponding *P*-values displayed as −log_10_ (*P*-value). Dot colours correspond to different lipid subclasses, and the dot size indicates significance. Each dot represents a lipid metabolite. Only lipids with *P <* .05 are shown. (*I*) Volcano plots showing the magnitude and significance of the altered lipid metabolites between AS and AAA mice. (*J*) Pie charts showing the composition of the altered lipid metabolites by lipid subclasses between AS and AAA mice. (*K*) Bar plots showing the degree of lipid metabolite changes between AS and AAA mice. (*L*) Dot plots showing log_2_ fold changes in lipid subclasses between AS and AAA mice, and the corresponding *P*-values displayed as −log_10_ (*P*-value). Dot colours correspond to different lipid subclasses, and the dot size indicates significance. Each dot represents a lipid metabolite. Only lipids with *P <* .05 are shown. AAA, abdominal aortic aneurysm; AS, atherosclerosis; BMP, bis(monoacylglycero) phosphate; CE, cholesterol esters; CER, ceramides; DAG, diglycerides; FFA, free fatty acids; HCER, hexosylceramides; LCER, lactosylceramides; LPC, lysophosphatidylcholines; LPE, lysophosphatidylethanolamines; LPG, lyso-phosphatidylglycerol; PC, phosphatidylcholines; PE, phosphatidylethanolamines; PG, phosphatidylglycerol; PI, phosphatidylinositol; PS, phosphatidylserine; SM, sphingomyelins; TAG, triglycerides; PCA, principal component analysis; ORO, Oil Red O; VIP, variable importance in projection

### Lipid metabolism differs between patients with atherosclerosis and abdominal aortic aneurysm

To verify our findings from animal experiments, we conducted lipidomic analyses on plasma samples from 363 individuals from Renji hospital (including 121 patients with documented CAD, 121 patients with documented AAA, and 121 age- and sex-matched healthy controls). The analysis identified 705 lipid metabolites, of which 313 were significantly elevated in CAD patients and 174 in AAA patients compared to controls (*Figure 2A*). In CAD patients, glycerolipids accounted for the largest proportion of the increased lipid metabolites (60.4%). In contrast, phosphoglycerides were the most abundant lipid class among the differential metabolites in AAA patients, accounting for 68.4% (*Figure 2B*). Consistent with the results from animal studies, further lipid subclass analysis revealed that triglycerides were the most significantly elevated lipid subclass in CAD, followed by diglycerides, while phosphatidylcholines were the most significantly increased lipid subclass in AAA, followed by phosphatidylethanolamines (*Figure 2C–F*). Direct comparison between CAD and AAA patients identified 135 differentially expressed lipid metabolites (100 upregulated and 35 downregulated in CAD relative to AAA) (*Figure 2G*), belonging to three lipid classes (glycerolipids, phosphoglycerides, and sterol lipids). Subclass analysis showed that triglycerides, diglycerides, and cholesterol ester exhibited significant increase in CAD. Conversely, phosphatidylcholines displayed the greatest increase in AAA, followed by lyso-phosphatidylethanolamines and monogalactosyldiacylglycerols (*Figure 2H–J*). The top five significantly upregulated and downregulated lipid metabolites in CAD compared to AAA are shown in *Figure 2K*. Importantly, when comparing lipidomic data from human cohorts with those from animal models, we observed a similar pattern of altered lipid metabolite subclasses in human vs mouse samples (*Figure 2L*). Overall, these human findings align with lipidomic analyses from animal models, underscoring the distinct lipidomic features characterizing AS and AAA.

**Figure 2:**
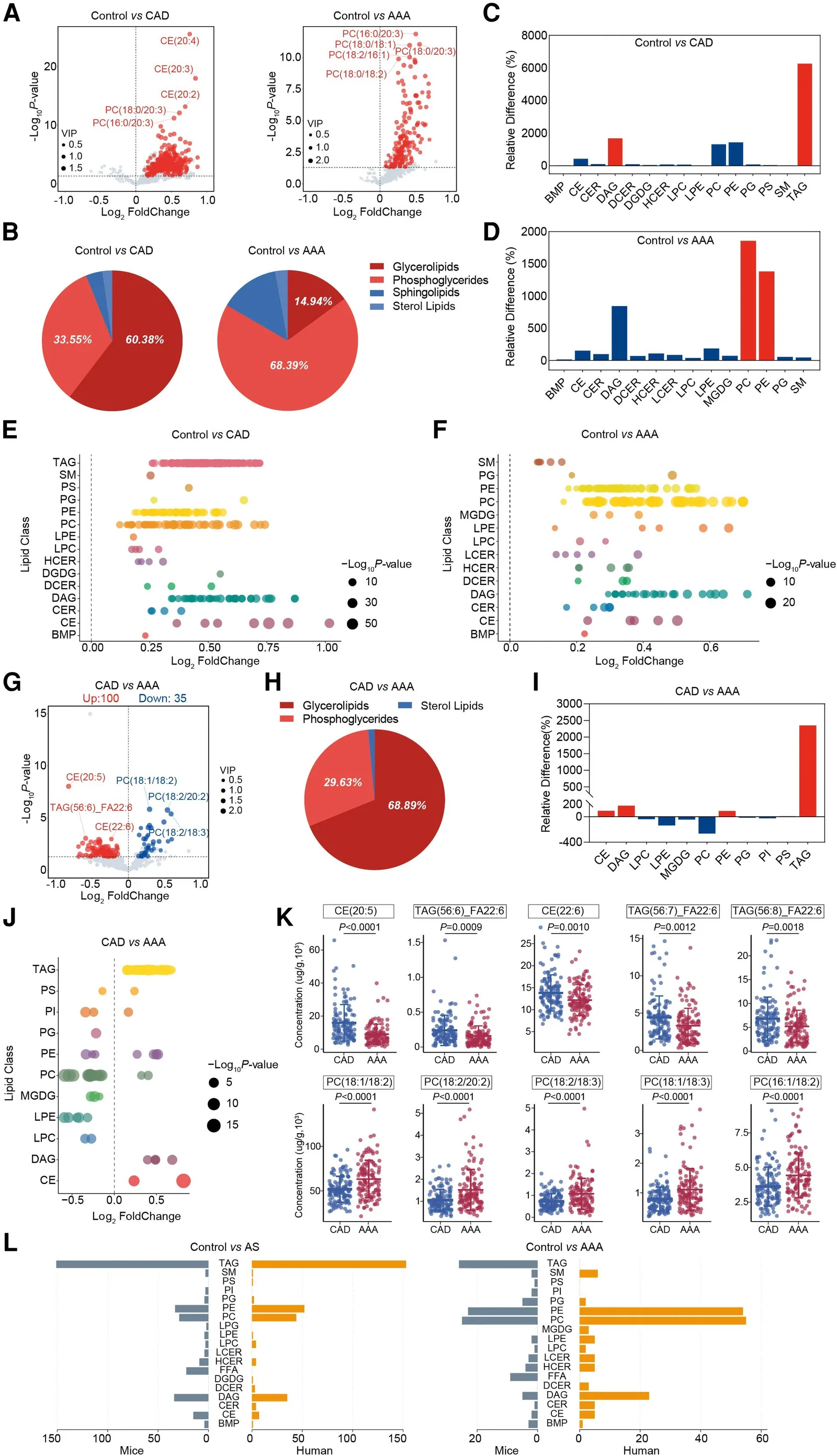
Lipid metabolism differs between patients with AS and AAA. (*A*) Volcano plots showing significant alterations in the lipidome of CAD and AAA groups compared to healthy control group (*n* = 121 per group). Red dots indicate upregulated lipid metabolites (VIP > 1 and *P* < .05). (*B*) Pie charts showing the composition of the altered lipid metabolites by lipid classes in CAD and AAA groups compared to healthy control group. (*C* and *D*) Bar plots showing the degree of lipid metabolite changes in CAD and AAA groups compared to the healthy control group. Red bars represent the top two most significantly altered lipid subclasses. (*E* and *F*) Dot plots showing log_2_ fold changes in lipid subclasses in CAD and AAA groups compared to healthy control group and the corresponding *P*-values displayed as −log_10_ (*P*-value). Dot colours correspond to different lipid subclasses, and the dot size indicates significance. Each dot represents a lipid metabolite. Only lipids with *P <* .05 are shown. (*G*) Volcano plots showing the magnitude and significance of the altered lipid metabolites between CAD and AAA patients. (*H*) Pie charts showing the composition of the altered lipid metabolites by lipid classes between CAD and AAA patients. (*I*) Bar plots showing the degree of lipid metabolite changes between CAD and AAA patients. Red bars represent upregulated lipid subclasses and blue bars represent downregulated lipid subclasses in CAD compared to AAA patients. (*J*) Dot plots of log_2_ fold changes in lipid subclasses between CAD and AAA patients and the corresponding *P*-values displayed as −log_10_ (*P*-value). Dot colours correspond to different lipid subclasses, and the dot size indicates significance. Each dot represents a lipid metabolite. Only lipids with *P <* .05 are shown. (*K*) Scatter plots showing the top 5 upregulated lipids and top 5 downregulated lipids in CAD compared to AAA patients. Data were analysed by Student’s *t*-test. (*L*) Comparison of differential lipid abundances between human and mouse sets. AAA, abdominal aortic aneurysm; BMP, bis (monoacylglycero) phosphate; CAD, coronary artery disease; CE, cholesterol esters; CER, ceramides; DAG, diglycerides; DCER, dihydroceramides; DGDG, digalactosyldiacylglycerol; FFA, free fatty acids; HCER, hexosylceramides; LCER, lactosylceramides; LPC, lysophosphatidylcholines; LPE, lysophosphatidylethanolamines; LPG, lyso-phosphatidylglycerol; MGDG, monogalactosyldiacylglycerol; PC, phosphatidylcholines; PE, phosphatidylethanolamines; PG, phosphatidylglycerol; PI, phosphatidylinositol; PS, phosphatidylserine; SM, sphingomyelins; TAG, triglycerides; FC, fold change; VIP, variable importance in projection

To further explore longitudinal associations between lipid traits and the risks of AS and AAA, we leveraged data from the UK Biobank study. Our analysis included 479 256 adults without CAD or AAA at baseline, with a median age of 57 years, 55.7% of whom were female. During a median follow-up of 14.8 years (IQR: 14.1–15.5 years), 22 675 incident CAD cases and 2159 incident AAA cases were identified. Consistently, the metabolic and proteomic data from the UK Biobank highlight the distinct roles of lipid metabolic disturbances in the further risk of AS and AAA (details in Supplementary data online, *Results*). Using both Cox proportional hazards model and propensity score matching adjusted analysis, the results showed strong positive associations of triglycerides, cholesterol, and fatty acid with the risk of CAD, while phosphoglycerides were negatively associated with the risk of AAA (details in Supplementary data online, *Results*, *Figures S3* and *S4*, and *Tables S3*–*S5*).

### Fatty acid metabolism is identified as the top altered lipid-related pathway contributing to the lipid metabolic differences between atherosclerosis and abdominal aortic aneurysm

To explore the mechanisms underlying the differences in lipid metabolism between AS and AAA, we performed high-throughput RNA sequencing (RNA-seq) on mouse aortas from wire injury-induced AS and the Ang II-induced AAA models. Principal component analysis of the transcriptomes showed a clear separation between the AS and AAA groups (*Figure 3A*). Differential gene expression analyses (fold change ≥ 2, *P <* .05) showed significant alterations in both AS and AAA compared to controls (*Figure 3B*). Gene Ontology (GO) enrichment analysis of differentially expressed genes (DEGs) indicated that both immune-related and inflammatory-related responses were upregulated in both AS and AAA (*Figure 3C* and *D*); however, pathways enriched among downregulated genes differed substantially between these two diseases: FA metabolic processes among lipid metabolism were predominantly downregulated in AS, while pathways related to cell adhesion and multicellular organism development were more significantly downregulated in AAA (*Figure 3C* and *D*). To gain deeper insights, we directly compared the transcriptomes of AS vs AAA (*Figure 3E*). Gene Ontology enrichment analysis revealed that lipid metabolic pathways relative to FA metabolism were the most significantly downregulated pathway in AS (*Figure 3F*), while pathways related to cell adhesion, extracellular matrix organization, and cell migration were most significantly suppressed in AAA (*Figure 3G*). Furthermore, metabolomic analyses of plasma samples demonstrated that FA biosynthesis was the top enriched pathway in AS, while suppression of FA metabolism was not seen in AAA (see Supplementary data online, *Figure S5A*–*D*). In contrast, phosphatidylethanolamines biosynthesis was the top enriched pathway in AAA (see Supplementary data online, *Figure S5E*). These findings suggest that FA metabolism is suppressed in AS, but not in AAA.

**Figure 3:**
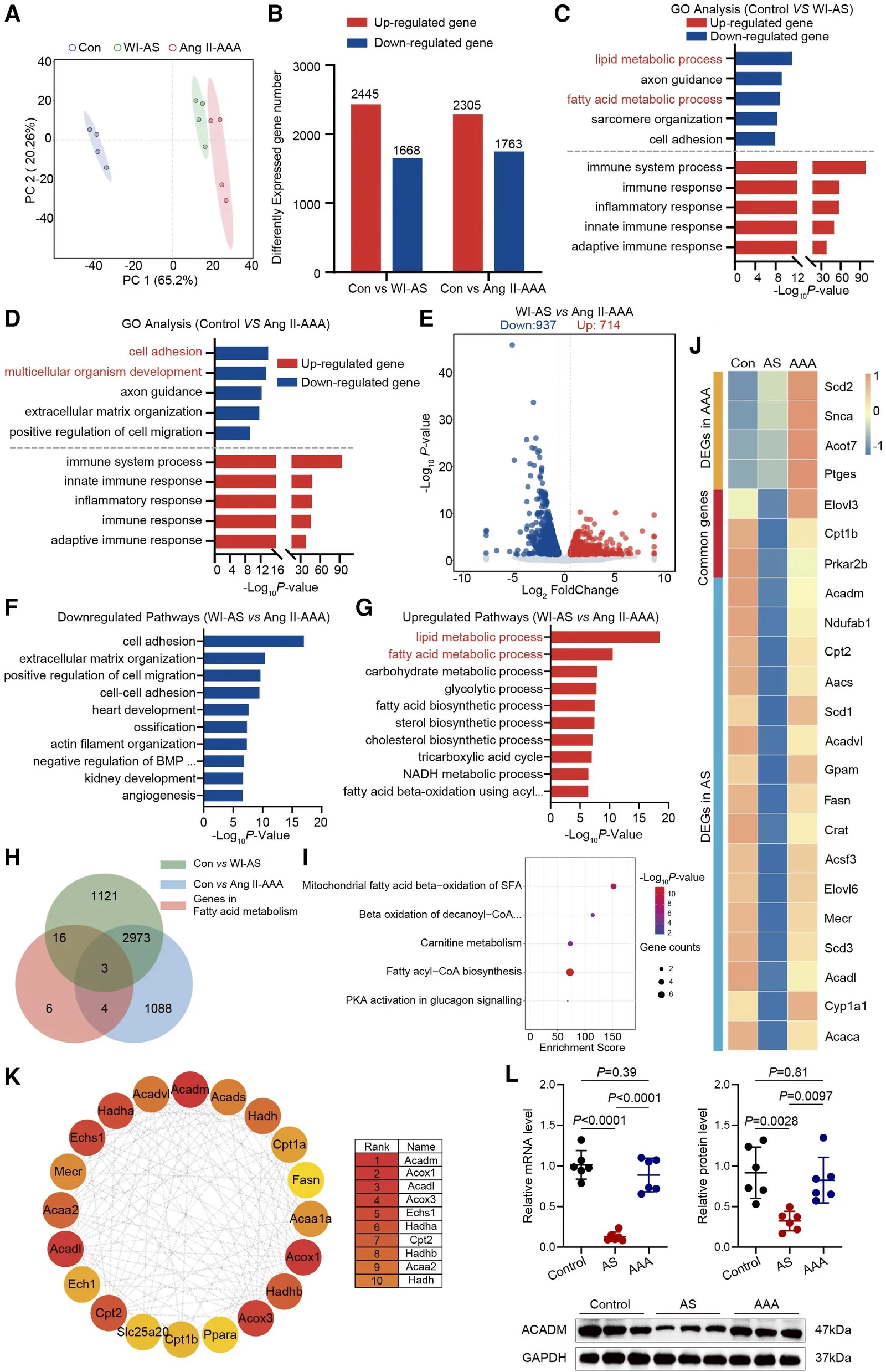
FA metabolism is identified as the top altered lipid-related pathway contributing to the lipid metabolic differences between AS and AAA. (*A*) Principal component analysis score plots of RNA-Seq data from mouse aortas among control, AS, and AAA groups (*n* = 4 per group). (*B*) Bar plots showing the number of DEGs in AS and AAA mice compared to controls. Differentially expressed genes were defined as genes with *P <* .05 and FC ≥ 2. (*C* and *D*) Bar plots showing the top 5 enriched GO biological process terms of the upregulated and downregulated DEGs in the (*C*) AS vs control and (*D*) AAA vs control comparisons. (*E*) Volcano plots showing the magnitude and significance of gene expression changes between AS and AAA mice. Differentially expressed genes were defined as *P <* .05 and FC ≥ 1.5. (*F* and *G*) Bar plots showing the top 10 enriched GO biological process terms of the upregulated and downregulated DEGs between AS and AAA mice. (*H*) Venn diagrams showing overlapping DEGs among the indicated groups. (*I*) Bubble charts showing the top 5 pathways enriched for DEGs within the ‘fatty acid metabolism process’ GO term. (*J*) Heatmaps showing the expression patterns of DEGs within the ‘fatty acid metabolism process’ GO term. (*K*) The protein–protein interaction analysis showing the top-ranked genes. (*L*) Top: Quantification of *Acadm* mRNA and ACADM protein expression measured by RT-qPCR and Western blot in mouse abdominal aortic segments (*n* = 6 per group). Data were analysed by one-way ANOVA test followed by Tukey’s *post hoc* test. Bottom: ACADM protein expression assessed by Western blot in mouse abdominal aortic segments. PCA, principal component analysis; AS, atherosclerosis; AAA, abdominal aortic aneurysm; DEGs, differentially expressed genes; FC, fold change; GO, Gene Ontology

To further validate the aforementioned findings, we conducted transcriptomic and metabolomic analyses using another HFD-induced AS model and another CaPO_4_-induced AAA model (see Supplementary data online, *Figure S6A* and *B*). Gene Ontology and Reactome enrichment analyses of DEGs confirmed that FA metabolism was significantly downregulated in HFD-induced AS (see Supplementary data online, *Figure S6C*–*E*) but not in CaPO_4_-induced AAA models (see Supplementary data online, *Figure S6F*–*H*). Metabolomic pathway enrichment analyses of plasma samples showed that FA biosynthesis was the top enriched pathway in HFD-induced AS, while ammonia recycling was the top enriched pathway in CaPO_4_-induced AAA (see Supplementary data online, *Figure S6I* and *J*). Overall, these multi-omics analyses consistently demonstrate the crucial role of downregulated FA metabolism in atherogenesis in a model-independent manner.

We further screened for genes involved in FA metabolism that were altered during AS and AAA progression (*Figure 3H*). Reactome pathway enrichment analysis highlighted FA β-oxidation (FAO) as the top downregulated pathway in AS mice (*Figure 3I*). The heatmap further illustrated differentially regulated genes associated with FA metabolism in AS and AAA (*Figure 3J*). Furthermore, protein–protein interaction network analysis identified *Acadm*, which encodes medium-chain acyl-CoA dehydrogenase (a key mitochondrial enzyme responsible for FAO),^28^ as a critical node in FA metabolism (*Figure 3K*). RT-qPCR and Western blot confirmed that both *Acadm* mRNA and ACADM protein levels were downregulated in AS mice but not in AAA (*Figure 3L*). To confirm the above findings, we analysed the human AS microarray dataset (GSE57691) from the Gene Expression Omnibus database. Differentially expressed genes visualized by Venn diagram revealed that four FA metabolism-related DEGs were co-expressed in both human and mouse AS: *Acadm*, *Cpt2*, *Acadl*, and *Ndufab1* (see Supplementary data online, *Figure S7A*). Among these four candidate genes, *Acadm* was the most significantly downregulated gene in the abdominal aortas of AS mice (see Supplementary data online, *Figure S7B*). The downregulation of *ACADM* mRNA and ACADM protein was further confirmed in human AS tissues by RT-qPCR and Western blot, whereas no significant change was observed in AAA tissues (see Supplementary data online, *Figure S7C* and *D*). Immunofluorescence confirmed that ACADM is predominantly expressed in aortic macrophages and that its expression is markedly reduced in AS but not in AAA (see Supplementary data online, *Figure S7E* and *F*). To further validate the downregulation of *Acadm* in macrophages, bone marrow–derived macrophages (BMDMs) were isolated from mice and treated with oxidized LDL (ox-LDL). Both *Acadm* mRNA and ACADM protein levels were significantly reduced in ox-LDL–treated BMDMs (see Supplementary data online, *Figure S7G* and *H*). *Acadm* deficiency results in the accumulation of carnitine derivatives (metabolic markers of impaired FAO), particularly octanoylcarnitine.^29,30^ We therefore measured acylcarnitine concentrations in both serum and vascular tissues from both mice and humans. Compared with controls, the levels of medium-chain acylcarnitines (i.e. octanoylcarnitine, hexanoylcarnitine, and decanoylcarnitine) were significantly elevated in AS, whereas no significant changes were observed in AAA (see Supplementary data online, *Figure S8A*–*F*). These findings provide compelling evidence that FA metabolism, particularly the FAO pathway, is the most significantly altered lipid-related pathway, contributing to the lipid metabolic differences between these two diseases.

### Lipid droplet clearance by lipid droplet autophagy-tethering compound treatment alleviates atherosclerosis but not abdominal aortic aneurysm in mice

FAO is the primary pathway for the degradation of FAs.^31^ Its impairment leads to the accumulation of excess FAs in the form of neutral lipids, predominantly triglycerides and cholesteryl ester, within lipid droplets (LDs).^31^ Lipid droplets regulate cellular FA levels through dynamic cycles of formation and breakdown.^32^ Therefore, understanding the role of LDs is important to gain deeper insights into FA metabolism in both AS and AAA. Based on the concept of ATTEC, we recently developed a novel LD-degrading strategy, LD·ATTEC, which tethers LDs to autophagosomes for autophagic degradation in several cells including adipocytes and embryonic fibroblasts.^33,34^ However, the effects of LD-degrading strategies in vascular diseases have never been investigated. Given the excessive accumulation of LDs in macrophages can lead to foam cell formation, we further investigated whether LD·ATTEC could reduce LDs in macrophages. We found that in macrophages, LD·ATTEC reduced LD accumulation (*Figure 4A–D*) by enhancing their degradation via autophagy (details in Supplementary data online, *Results* and *Figure S9*).

**Figure 4:**
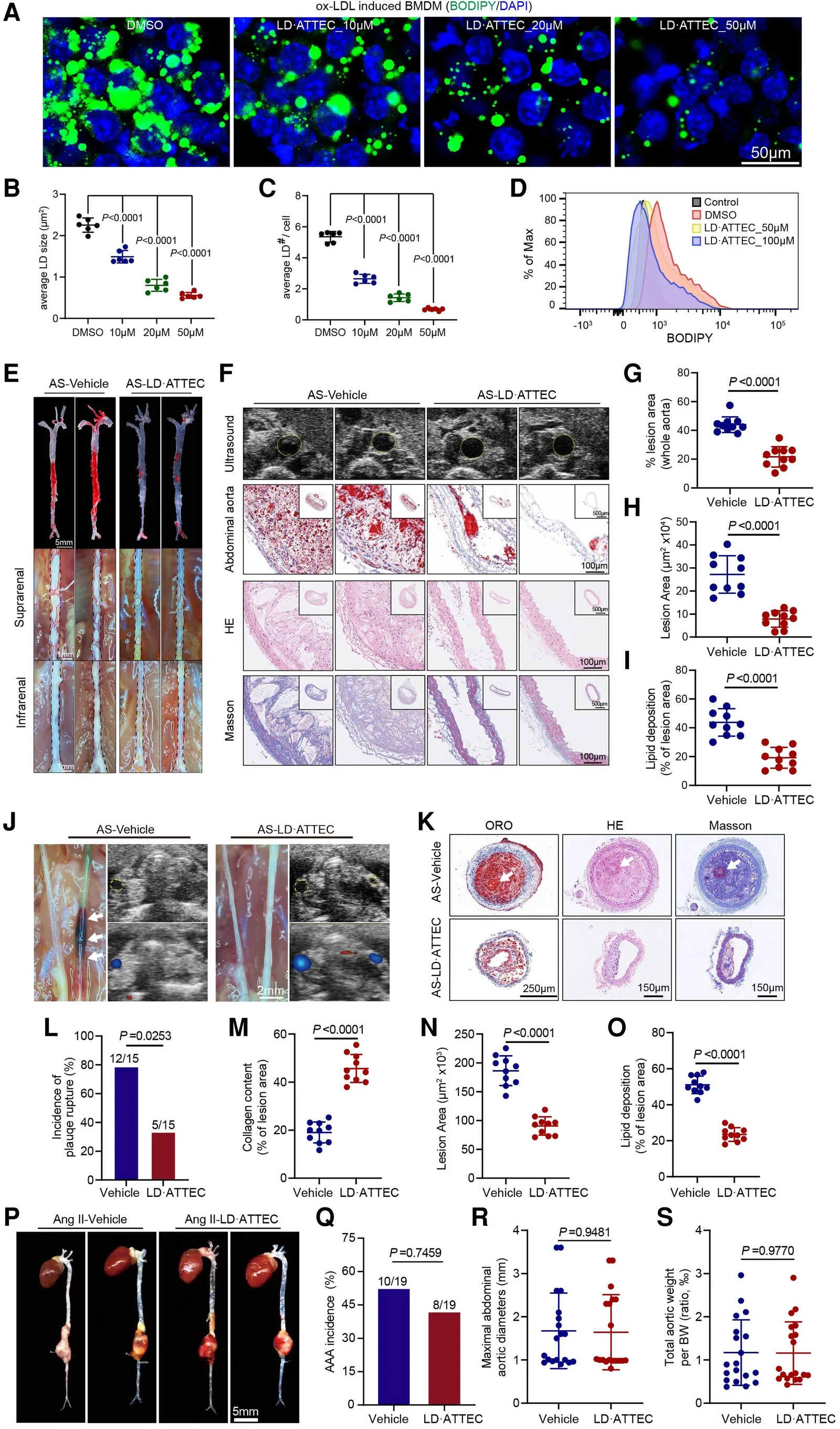
LD·ATTEC treatment reduces LDs and alleviates AS in mice. (*A–C*) Representative images and quantifications of LDs induced by treatment with 100 µg/mL ox-LDL for 24 h in BMDMs treated with the indicated compounds (*n* = 6 independent experiments). Data were analysed using one-way ANOVA, followed by Tukey’s *post hoc* test. (*D*) Flow cytometry analysis of LDs stained with BODIPY 493/503 in BMDMs pre-treated with 100 µg/mL ox-LDL for 24 h and subsequently treated with the indicated compounds. (*E*) Representative images of the ORO-stained whole aortas, and macroscopic features of wire injury-induced AS in abdominal aortas. (*F*) Representative images of abdominal aortas visualized using MUI (top), ORO-stained crossed-sections of abdominal aortas (middle), and crossed-sections stained with HE and Masson of abdominal aortas (bottom). (*G–I*) Quantification of the ORO-positive areas in the whole aortas (percentage of whole aortas; *n* = 10 per group) (*G*), lesion area volume of abdominal aorta sections (×10^4^ μm^2^; *n* = 10 per group) (*H*), and lipid deposition (percentage of lesion area; *n* = 10 per group) (*I*). Data were analysed by Student’s *t*-test. (*J*) Representative images of the carotid arteries and MUI visualizations. White arrows indicate thrombi. (*K*) Representative photomicrographs of carotid frozen sections stained with ORO, HE, and Masson. White arrows indicate thrombi. (*L–O*) Quantification of the incidence of plaque rupture (percentage; *n* = 15 per group) (*L*); lesion area volume (×10^3^ μm^2^; *n* = 10 per group) (*M*); lipid deposition (percentage of lesion area; *n* = 10 per group) (*N*); and collagen content (percentage of lesion area; *n* = 10 per group) (*O*). Data were analysed using Fisher’s exact test (*I*) or Student’s *t*-test (*J*–*L*). (*P*) Representative images of the macroscopic features of AAA formation in the indicated groups. (*Q*) The incidence of Ang II-induced AAA in the indicated groups. Data were analysed using Fisher’s exact test (*n* = 19 per group). (*R* and *S*) Quantification of the maximal diameter of abdominal aortas measured by a Digital Vernier Caliper and total aortic weight/BW in the indicated groups (*n* = 19 per group). Data were analysed by Mann–Whitney *U* test (*R* and *S*). AAA, abdominal aortic aneurysm; Ang II, angiotensin II; AS, atherosclerosis; BMDM, bone marrow–derived macrophage; BW, body weight; LDs, lipid droplets; HE staining, haematoxylin and eosin staining; MUI, micro-ultrasound imaging; ORO, Oil Red O; ox-LDL, oxidized LDL; Masson, Masson’s trichrome

To clarify the different pathological roles of LDs in AS and AAA, we administered LD·ATTEC to mouse models of these two diseases. We first examined LD accumulation during the progression of AS and AAA in mice and observed a more pronounced accumulation of LDs in the lesion area of AS compared to AAA (see Supplementary data online, *Figure S10A*). Next, we evaluated the effects of LD·ATTEC on blood pressure and plasma lipid levels. Lipid droplet autophagy-tethering compound treatment significantly lowered plasma lipid levels of triglycerides, total cholesterol, and LDL cholesterol in both AS and AAA, without affecting blood pressure (see Supplementary data online, *Figure S10B*–*E*). We then evaluated the therapeutic efficacy of LD·ATTEC in AS mice. Lipid droplet autophagy-tethering compound treatment significantly reduced atherosclerotic lesions, as evidenced by Oil Red O staining of abdominal aortas and *in vivo* micro-ultrasound imaging (MUI) (*Figure 4E* and *F*). Histological analysis revealed that LD·ATTEC reduced plaque area and intraplaque lipid contents (*Figure 4G–I*).

Given that vulnerable plaques are associated with a high risk of acute cardiovascular events in humans, we further generated a rupture-prone vulnerable plaque model, as we previously described.^35–37^ Treatment with LD·ATTEC markedly reduced carotid plaque burden (*Figure 4J* and *K*). Moreover, LD·ATTEC treatment significantly decreased the incidence of plaque rupture, and reduced atherosclerotic area and lesional lipid deposition (*Figure 4L–O*). These results suggest that LD·ATTEC reduced lipid accumulation and enhanced the stability of vulnerable atherosclerotic plaques.

To determine whether LD·ATTEC had similar therapeutic effects in AAA, we administered LD·ATTEC to AAA mice. However, LD·ATTEC treatment failed to ameliorate AAA formation, with no significant differences observed in AAA incidence, maximal suprarenal aortic diameter, or total aortic weight/body weight ratio (*Figure 4P–S*). Similar results were observed by visualization of the MUI (see Supplementary data online, *Figure S11A* and *B*). Histological analysis showed that LD·ATTEC treatment significantly decreased lipid deposition without affecting collagen contents (see Supplementary data online, *Figure S11C* and *D*). Collectively, although LD·ATTEC treatment successfully eliminated LDs in both AS and AAA mouse models, it only alleviated the pathological progression of AS (but not AAA), suggesting distinct roles of LDs in the pathogenesis of AS vs AAA.

### Lipid droplet clearance by lipid droplet autophagy-tethering compound treatment restores FA metabolism in atherosclerosis but not in abdominal aortic aneurysm mice

To explore the mechanisms underlying the differential pathogenic role of LDs in AS and AAA, we performed transcriptomic and metabolomic analyses to investigate the impact of LD·ATTEC on these two vascular diseases (*Figure 5A*). Differentially expressed genes are presented in *Figure 5B* and *C*. Key genes regulated by LD·ATTEC were identified by determining the intersection of the DEGs (*Figure 5D*). Gene Ontology analysis revealed that LD·ATTEC treatment upregulated pathways primarily associated with lipid metabolism related to FA metabolism in AS-LD·ATTEC group, while downregulating pathways related to inflammatory and immune responses (*Figure 5E*). In contrast, the AAA-LD·ATTEC group did not exhibit any significant changes in lipid metabolism-related pathways (*Figure 5F*). Reactome analysis of FA metabolism-related genes identified FAO as the most significantly enriched pathway in AS, whereas no such enrichment was observed in AAA after LD·ATTEC treatment (*Figure 5G*). Specifically, LD·ATTEC significantly increased the expression of FA metabolism-related genes in AS but did not significantly alter these genes in AAA (*Figure 5H*). Further RT-qPCR and Western blot analyses validated the significant upregulation of *Acadm* mRNA and ACADM protein following LD·ATTEC treatment in AS mice but not in AAA mice (*Figure 5I* and *J*).

**Figure 5:**
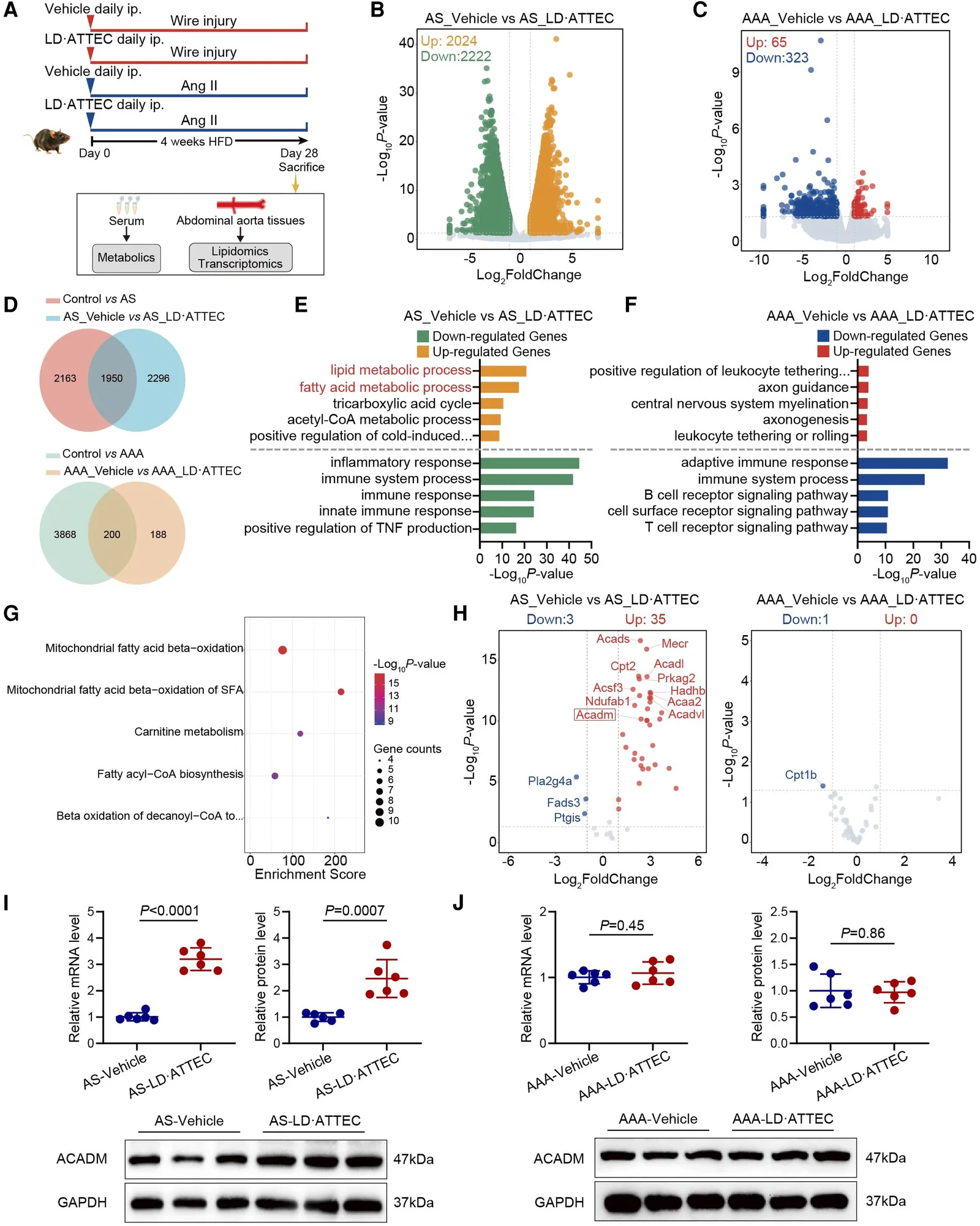
LD clearance by LD·ATTEC treatment induces different transcriptomic changes in AS and AAA mice. (*A*) Schematic overview of the animal experimental design. (*B* and *C*) Volcano plots showing log_2_ fold change and *P*-value of individual genes in mouse aortas (*n* = 4 mice per group). (*B*) Comparison between the AS_Vehicle and AS_LD·ATTEC groups. (*C*) Comparison between the AAA_Vehicle and AAA_LD·ATTEC groups. (*D*) Venn diagrams showing the overlap of genes with fold change ≥ 2 among the indicated groups. (*E* and *F*) Bar plots showing the top 5 enriched GO biological processes of upregulated and downregulated DEGs. (*E*) Comparison between the AS_Vehicle and AS_LD·ATTEC groups. (*F*) Comparison between the AAA_Vehicle and AAA_LD·ATTEC groups. (*G*) Bubble plot showing the top 5 pathways enriched for genes within ‘Fatty acid metabolic process’ GO term. (*H*) Volcano plots depicting DEGs involved in fatty acid metabolism following LD·ATTEC treatment. (*I*) Top: Quantification of *Acadm* mRNA and ACADM protein expression measured by RT-qPCR and Western blot in abdominal aortic segments of AS mice treated with vehicle or LD·ATTEC (*n* = 6 per group). Data were analysed by Student’s *t*-test. Bottom: ACADM protein expression assessed by Western blot in mouse abdominal aortic segments. (*J*) Top: Quantification of *Acadm* mRNA and ACADM protein expression measured by RT-qPCR and Western blot in abdominal aortic segments of AAA mice treated with vehicle or LD·ATTEC (*n* = 6 per group). Data were analysed by Student’s *t*-test. Bottom: ACADM protein expression assessed by Western blot in mouse abdominal aortic segments. Ang II, angiotensin II; AS, atherosclerosis; AAA, abdominal aortic aneurysm; GO, gene ontology

To further investigate the effects of LD·ATTEC on metabolism, we performed metabolomic analysis (see Supplementary data online, *Figure S12A*). Lipid droplet autophagy-tethering compound administration predominantly impacted lipid metabolism in both AS and AAA (see Supplementary data online, *Figure S12B*), with different pattern. The differences in metabolite expression are shown in the volcano plot (see Supplementary data online, *Figure S12C*), with the top 20 differentially expressed metabolites displayed in the heatmap (see Supplementary data online, *Figure S12D*). Notably, acylcarnitines (the metabolite produced during FAO and accumulating upon the disrupted FAO pathway^30^) were significantly reduced in plasma following LD·ATTEC treatment (see Supplementary data online, *Figure S12E*). In summary, transcriptomic and metabolomic analyses revealed that LD·ATTEC restored FA metabolism and FAO pathway in AS, but did not show significant changes in AAA.

To achieve a better understanding of lipid metabolic alterations, we further performed lipidomic analysis on abdominal aortic samples from LD·ATTEC-treated AS mice. Principal component analysis revealed distinct lipidomic signatures between the LD·ATTEC-treated and vehicle-treated AS mice (see Supplementary data online, *Figure S13A*). Lipid droplet autophagy-tethering compound treatment reduced the total lipid contents in the aortas by 67.6 ± 14.3% (calculated based on the sum of detected lipids) in AS mice. A total of 215 lipid metabolites were significantly downregulated in the LD·ATTEC-treated AS mice compared to the vehicle-treated controls (see Supplementary data online, *Figure S13B*). Glycerolipids were the most affected lipid category (see Supplementary data online, *Figure S13C*). Of the 23 lipid subclasses detected, 11 displayed significant changes with LD·ATTEC treatment; the top 4 changed lipid subclasses were triglycerides, diglycerides, cholesteryl esters, and free fatty acids (see Supplementary data online, *Figure S13D* and *E*). Specifically, LD·ATTEC reduced the levels of neutral lipids (e.g. triglycerides, diglycerides, cholesterol esters) and FFA to near-control levels (see Supplementary data online, *Figure S13F*–*I*).

### Acadm overexpression represses lipid droplet formation and mitigates atherosclerosis but not abdominal aortic aneurysm in mice

*Acadm* serves as a key enzyme in mitochondrial FAO and plays an essential role in regulating FA metabolism.^38^ However, the functional role of *Acadm* in the pathogenesis of vascular diseases including AS and AAA has never been investigated. Our aforementioned findings identified *Acadm* as a hub DEG among the FA metabolism-related genes between AS and AAA. Therefore, we further explored its potential role in AS and AAA formation. First, we overexpressed *Acadm* in BMDM via lentivirus infection (*Figure 6A*). *Acadm* overexpression significantly reduced LD accumulation in ox-LDL-treated BMDM (*Figure 6B*). To further investigate the role of *Acadm* in the development of AS and AAA *in vivo*, we generated myeloid-specific *Acadm* overexpressing mice, which were subjected to AS or AAA model induction (see Supplementary data online, *Figure S14*; *Figure 6C*). *Acadm* overexpression significantly reduced atherosclerotic lesions compared to the controls in the AS models (*Figure 6D–H*), but failed to attenuate AAA progression in the AAA models (*Figure 6I–P*). The protective role of *Acadm* against AS found in animal models was further supported by the human whole-exome sequencing data from the UK Biobank, which demonstrated that loss-of-function mutations in *ACADM* were associated with increased AS burden (details in Supplementary data online, *Results*, *Figure S15 and Table S6*). Overall, the key FAO metabolic enzyme *Acadm* exerts differential effects in AS vs AAA, further supporting the distinct roles of FA metabolism in the pathogenesis of these two diseases.

**Figure 6:**
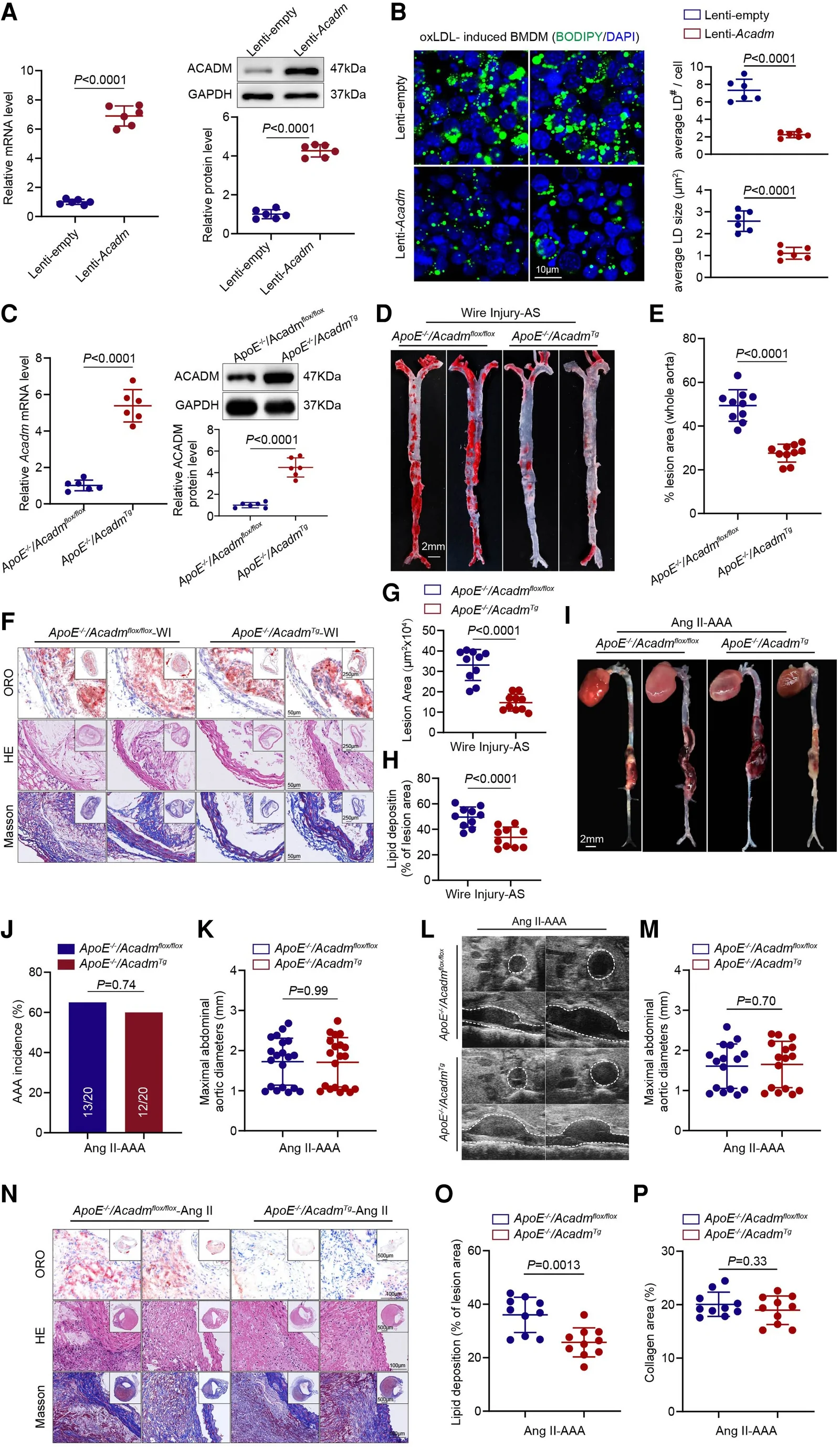
Myeloid-specific *Acadm* overexpression represses LD formation and mitigates AS but not AAA in mice. (*A*) BMDMs were infected with the lentiviral empty vectors or the *Acadm*-overexpression lentiviral vectors. The overexpression efficiency of *Acadm* expression was determined by RT-qPCR and Western blot analyses. Left: Quantification of *Acadm* mRNA expression in BMDMs measured by RT-qPCR (*n* = 6 per group). Right: Representative images of Western blot for ACADM in BMDMs and quantification for Western blot analysis (*n* = 6 per group). Data were analysed by Student’s *t*-test. (*B*) BMDMs were infected and treated with ox-LDL (100 µg/mL, 24 h). Representative images and quantification of BODIPY493/503 staining of the LDs in the indicated groups. The LD number per cell and average LD size were analysed (*n* = 6 independent experiments). (*C*) BMDMs were isolated from myeloid-specific *Acadm* overexpression mice. Left: Quantification of *Acadm* mRNA expression in BMDMs measured by RT-qPCR (*n* = 6 per group). Right: Representative images of Western blot for ACADM in BMDMs and quantification for Western blot analysis (*n* = 6 per group). Data were analysed by Student’s *t*-test. (*D*) Representative images of the ORO-stained whole aortas. (*E*) Quantification of the ORO-positive areas in the whole aortas (percentage of whole aortas; *n* = 10 per group). Data were analysed by Student’s *t*-test. (*F*) Representative images of histological staining (ORO staining, HE staining, and Masson staining) of crossed-sections of abdominal aortas in the indicated groups. (*G* and *H*) lesion area volume of abdominal aorta sections (×10^4^ μm^2^; *n* = 10 per group) (*G*) and lipid deposition (percentage of lesion area; *n* = 10 per group) (*H*). Data were analysed by Student’s *t*-test. (*I*) Representative images of the macroscopic features of AAA formation in the indicated groups. (*J*) The incidence of Ang II-induced AAA in the indicated groups (*n* = 20 per group). Data were analysed using Fisher’s exact test. (*K*) Quantification of the maximal diameter of abdominal aortas by a Digital Vernier Caliper in the indicated groups (*n* = 20 per group). Data were analysed by Mann–Whitney *U* test. (*L* and *M*) Representative Doppler ultrasound images and quantification of the maximal diameter of abdominal aortas in the indicated groups (*n* = 14 per group). Data were analysed by Mann–Whitney *U* test. (*N*) Representative images of histological staining (ORO staining, HE staining, and Masson staining) of crossed-sections of abdominal aortas in indicated groups. (*O* and *P*) Quantification of lipid deposition (percentage of lesion area, *n* = 10 per group) (*O*) and collagen content (percentage of lesion area, *n* = 10 per group) (*P*). Data were analysed by Student’s *t*-test. AS, atherosclerosis; AAA, abdominal aortic aneurysm; Ang II, angiotensin II; BMDM, bone marrow–derived macrophage; ox-LDL, oxidized LDL; LD, lipid droplet; WI, wire injury; ORO, Oil Red O; HE, haematoxylin and eosin; Masson, Masson’s trichrome

*Acadm* deficiency has been reported to induce the accumulation of intracellular lipotoxic intermediates (e.g. FFA) generated during impaired FAO, thereby provoking mitochondrial dysfunction.^39^ To further elucidate the underlying mechanism, we performed both *Acadm* loss-of-function and gain-of-function experiments in BMDMs. *Acadm* deficiency by *Acadm*-knockdown lentivirus transduction increased intracellular total FFAs, accompanied by decreased acetyl-CoA and free-CoA pools (*Figure 7A*). Oxygen consumption analysis revealed that *Acadm* knockdown significantly decreased basal, maximal, and ATP-coupled mitochondrial oxygen consumption rates (OCRs) (*Figure 7B* and *C*). Moreover, *Acadm* knockdown disrupted mitochondrial membrane potential and increased mitochondrial reactive oxygen species (mtROS) (*Figure 7D*). To determine whether the mitochondrial dysfunction resulted from lipotoxic metabolite accumulation, BMDMs were treated with 2.5 μmol/L etomoxir, a pharmacological inhibitor that limits mitochondrial fatty acid uptake. Etomoxir effectively rescued the mitochondrial defects caused by *Acadm* depletion, restoring mitochondrial respiratory capacity and membrane potential, and reducing mtROS levels (*Figure 7E–J*). To further validate the protective effects of *Acadm*, BMDMs were transduced with *Acadm*-overexpression or control lentivirus and subsequently exposed to ox-LDL. *Acadm* overexpression restored lipid metabolic balance, as indicated by reduced intracellular lipotoxic metabolites FFAs, accompanied by increased acetyl-CoA and free-CoA levels (see Supplementary data online, *Figure S16A*). *Acadm* overexpression markedly improved mitochondrial respiration, membrane potential, and reduced oxidative stress (see Supplementary data online, *Figure S16B*–*I*). Collectively, these findings indicate that *Acadm* preserves mitochondrial homeostasis primarily by preventing the intracellular accumulation of lipotoxic metabolites.

**Figure 7:**
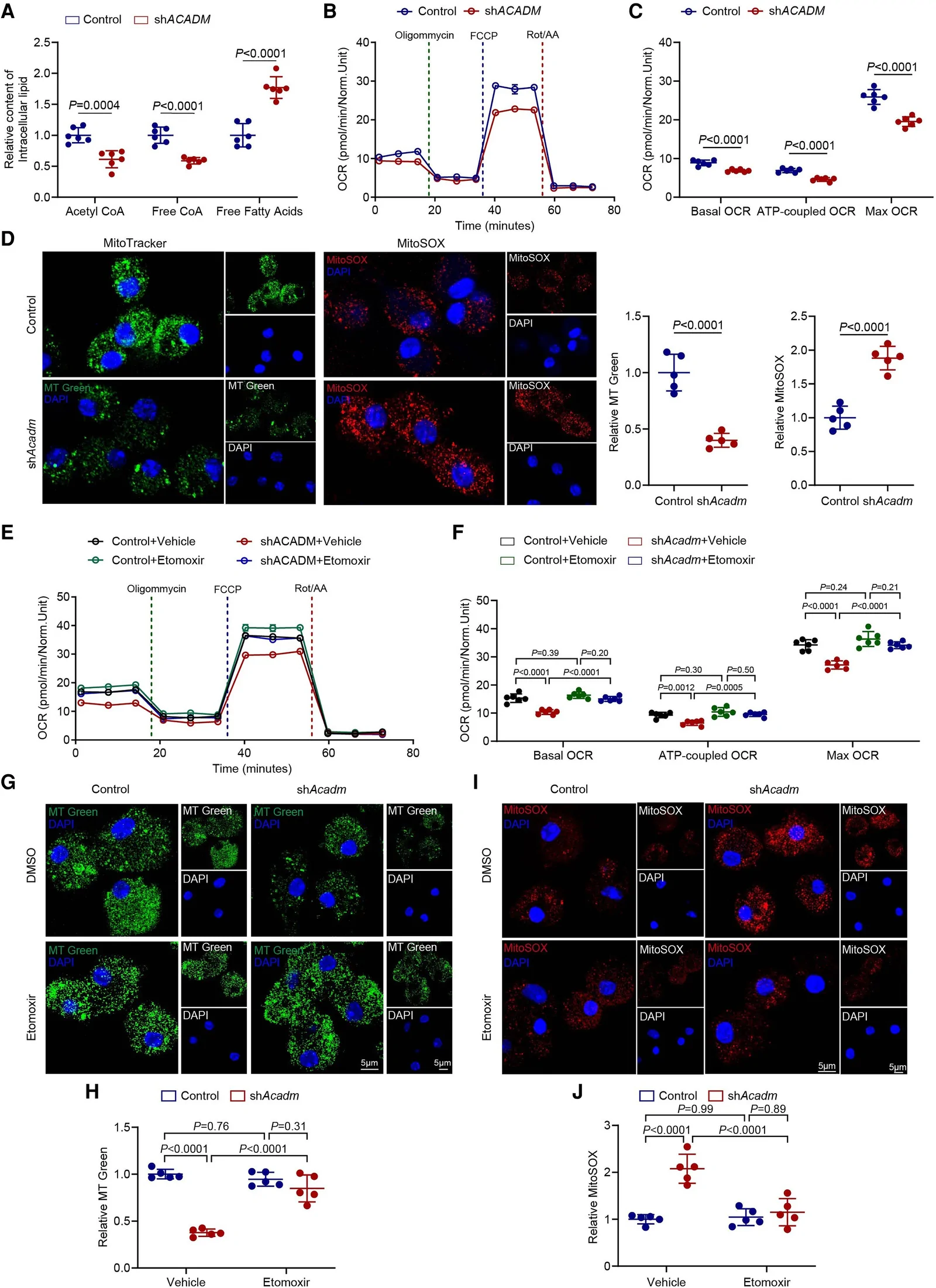
*Acadm* deficiency disrupts mitochondrial function by inducing lipotoxic metabolite accumulation in BMDMs. (*A*) Intracellular acetyl-CoA, free-CoA, and free fatty acid levels in BMDMs after *Acadm* knockdown (*n* = 6 independent experiments). Data were analysed by Student’s *t*-test. (*B*) Summarized OCR tracings in BMDMs transduced with *Acadm*-knockdown or control lentivirus (*n* = 6 independent experiments). (*C*) Quantification of basal, maximal, and ATP-coupled OCRs in indicated groups (*n* = 6 independent experiments). Data were analysed by Student’s *t*-test. (*D*) Representative images and quantitative analysis of mitochondrial membrane potential (MitoTracker Green, green) and mitochondrial ROS levels (MitoSOX, red) in control and *Acadm*-knockdown BMDMs (*n* = 5 independent experiments). Data were analysed by Student’s *t*-test. (*E*) Summarized OCR tracings in BMDMs transduced with *Acadm*-knockdown or control lentivirus, followed by etomoxir treatment (*n* = 6 independent experiments). (*F*) Quantification of basal, maximal, and ATP-coupled OCRs in indicated groups (*n* = 6 independent experiments). Data were analysed by two-way ANOVA followed by Tukey’s *post hoc* test. (*G*) Representative images of mitochondrial membrane potential assessed by MitoTracker Green staining in control and sh*Acadm*-transduced BMDMs treated with vehicle (DMSO) or etomoxir (2.5 μmol/L). (*H*) Quantification of MitoTracker Green staining (*n* = 5 independent experiments). (*I*) Representative images of mitochondrial ROS levels assessed by MitoSOX staining in control and sh*Acadm* BMDMs treated as in (*G*). (*J*) Quantification of MitoSOX staining (*n* = 5 independent experiments). Data were analysed by two-way ANOVA followed by Tukey’s *post hoc* test. ATP, adenosine triphosphate; BMDM, bone marrow–derived macrophage; OCR, oxygen consumption rate; ROS, reactive oxygen species

### Construction of machine learning predictive models based on lipid metabolic features

The above multi-omics and validation studies in human and preclinical animal models revealed different lipid metabolic features in AS vs AAA. Thus, we sought to further establish disease prediction models based on their distinct lipid metabolic features. Both AS and AAA could lead to life-threatening conditions. Commonly used biomarkers such as troponin, myoglobin, and creatine kinase are specific to acute myocardial infarction, while most AAA patients remain asymptomatic until lethal rupture.^19,40^ Given the distinct roles of lipid metabolism in AS and AAA, predictive models based on lipid features may provide unique advantages.

We first developed predictive models for CAD vs healthy controls (HC) as well as those for AAA vs HC based on our clinical lipidomic data using machine learning algorithms. Participants were randomized in a ratio of 7:3 into training and validation cohorts.^41^ We screened 13 significantly differential lipid metabolites in CAD patients and 25 metabolites in AAA patients in their respective training sets using a combination of *t*-test, least absolute shrinkage and selection operator (LASSO) regression, and partial least squares discriminant analysis (PLS-DA). Then, we developed logistic regression models using selected lipidomic features, adjusted for age, sex, and BMI. The area under receiver operating characteristic curve (AUC) for CAD vs HC model in the training cohort was 0.974 (95% CI 0.954–0.994), while the AUC for the AAA vs HC model in the training cohort was 0.913 (95% CI 0.870–0.956) (Supplementary data online, *Table S7*). In CAD, CE(20:4) showed the strongest association, followed by TAG(52:5), FA(20:3), and CE(20:3) (see Supplementary data online, *Figure S17A*). In contrast, for AAA, PC(16:0/20:4) exhibited the highest association, followed by PC(16:0/18:0), PC(18:2/18:3), PC(18:1/16:1), LPC(20:1), and LPE(18:0) (see Supplementary data online, *Figure S18A*). To enhance model interpretability, nomograms were constructed to visualize the predictive influence of lipid metabolites (see Supplementary data online, *Figures S17B* and *S18B*). Calibration curves demonstrated excellent concordance between predicted and observed risks (see Supplementary data online, *Figures S17C* and *S18C*). In the validation cohort, the AUC value for CAD vs HC was 0.832 (95% CI 0.741–0.923) and the AUC value for AAA vs HC was 0.789 (95% CI 0.734–0.929). To validate the stability of screened lipid metabolites for disease identification, we further constructed four additional machine learning models, including random forest (RF), support vector machines (SVM), extreme gradient boosting (XGBoost), and the Super Learner ensemble model. All models exhibited AUCs greater than 0.75 in the validation cohort (see Supplementary data online, *Figures S17D* and *S18D*). Supplementary data online, *Figure S17E* summarized the evaluation metrics—including accuracy, sensitivity, specificity, precision, F1 scores, and AUROCs—of the four models in the CAD vs HC validation cohort, while Supplementary data online, *Figure S18E* displayed those metrics of the four models in AAA vs HC validation cohort. Additionally, we measured the abundance of key lipids with significant coefficients in the logistic regression analysis for the CAD vs HC model (see Supplementary data online, *Figure S17F*) and the AAA vs HC model (see Supplementary data online, *Figure S18F*).

Next, we developed predictive models to differentiate between AS and AAA based on lipidomic features using machine learning algorithms. We identified 20 differential lipid metabolites between patients with CAD and AAA for model construction. The AUC of the logistic regression model using selected lipidomic features was 0.968 (95% CI 0.946–0.990) in the training set (*n* = 169) and 0.841 (95% CI 0.747–0.935) in the validation set (*n* = 73). The forest plot revealed that patients with CAD had higher levels of CE(20:2) and CE(20:4), whereas patients with AAA showed higher levels of PC(16:0/18:3) and PC(18:0/18:2) (*Figure 8A*). The nomogram constructed with selected lipid metabolites effectively distinguished CAD from AAA (*Figure 8B*). Furthermore, calibration curves demonstrated excellent agreement between predicted and observed risks (*Figure 8C*). Area under receiver operating characteristic curve values for all four models (logistic regression, RF, SVM, and XGBoost) exceeded 0.8 in the validation cohort (*Figure 8D*), confirming the robustness of the classifiers. Other evaluation metrics, including accuracy, sensitivity, specificity, precision, and F1 scores, are shown in *Figure 8E*. Finally, we examined the concentrations of these key lipids with significant coefficients in the logistic regression analysis and found that patients with CAD had significantly higher concentrations of cholesterol esters, whereas the phosphatidylcholine concentration was increased in patients with AAA (*Figure 8F*).

**Figure 8:**
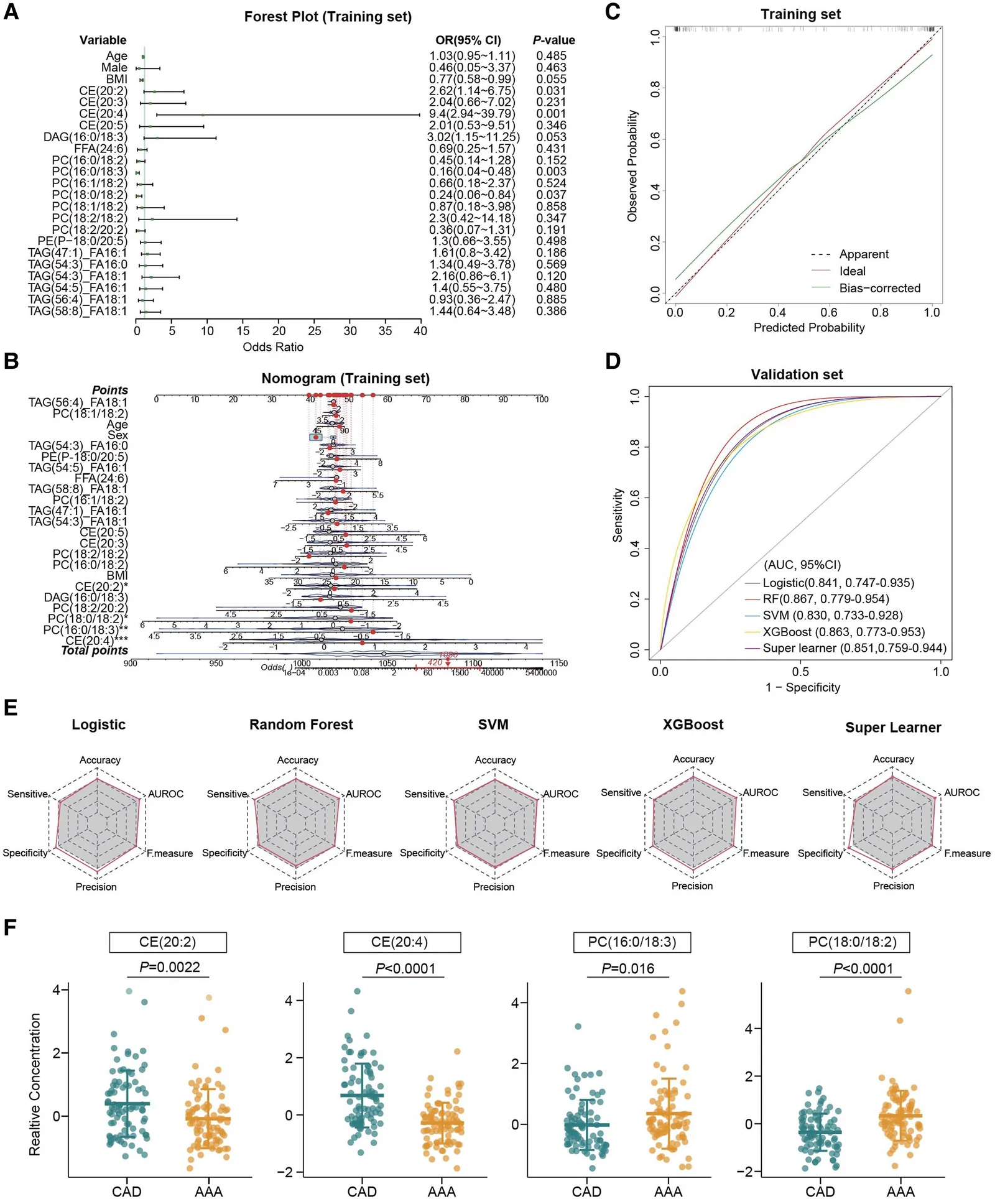
The establishment of machine learning model utilizing lipidomic features differentiating CAD *vs* AAA. (*A*) OR values of predictor variables and their 95% confidence intervals in the logistic model incorporating lipidomic features for CAD vs AAA in the training cohort (*n* = 169). (*B*) Nomograms constructed based on the training cohort for CAD vs AAA. (*C*) Calibration curves of the logistic regression analysis in the training cohort for CAD vs AAA. Calibration intercept = 0.001 (SE 0.288, *P* > .99); calibration slope = 1.004 (SE 0.170, *P* = .979). Joint test of intercept = 0 and slope = 1: χ^2^ = 0.001 (df = 2, *P* > .99). No statistically significant deviation from ideal calibration was observed. (*D*) ROC curve for four predictive models in the validation cohort (*n* = 73) for CAD vs AAA. (*E*) Radar plots of evaluation metrics for machine learning predictive models for CAD vs AAA. (*F*) Boxplots of concentration of the identified key lipid metabolites with significant coefficients in the logistic regression analysis in the indicated group. Data were analysed by Student’s *t*-test. AAA, abdominal aortic aneurysm; AS, atherosclerosis; AUC, area under receiver operating characteristic curve; CAD, coronary artery disease; CE, cholesterol esters; OR, odds ratio; PC, phosphatidylcholines; PE, phosphatidylethanolamines; ROC, receiver operating characteristic curve; TAG, triglycerides; SVM, support vector machine; XGBoost, extreme gradient boosting

## Discussion

To the best of our knowledge, this work is the first to systematically delineate the lipid metabolic differences and associated mechanisms in AS vs AAA by integrating multi-omics analyses in both animal studies and human cohorts (*Structured Graphical Abstract*). The novel contributions included the following. First, we identified differential lipid metabolic features between AS and AAA through lipidomic analyses in both mouse and human samples. Our findings revealed that neutral lipids (e.g. triglycerides and cholesterol esters) were significantly enriched in AS, while phosphoglycerides predominated in AAA. Consistently, large-scale longitudinal data from the UK Biobank demonstrated that triglycerides, cholesterol, and fatty acid were positively associated with incident CAD, whereas phosphoglycerides were inversely associated with incident AAA. Further mechanistic studies identified FA metabolism, centred on *Acadm*-mediated FAO pathway, as the most significantly altered lipid-related pathway contributing to the lipid metabolic differences between these two diseases. Selective clearance of LDs using LD·ATTEC treatment restored FA metabolism and inhibited AS progression, whereas no significant change was observed in AAA. Finally, we constructed machine learning predictive models based on lipidomic signatures enabling accurate differentiation between AS and AAA. Collectively, our study provides compelling evidence that AS and AAA are distinct diseases from perspective of lipid metabolism, highlighting the need for tailored therapeutic strategies for these two diseases (see Supplementary data online, *Figure S19*).

Examination of human biopsies identified concurrent of AAA and AS, leading to the historical assumption that AAA results from AS.^42^ However, emerging evidence indicates that AAA is more closely linked to generalized arteriomegaly rather than advanced AS.^43^ Moreover, genes differentially expressed in AAA and AS were distinct, implying the diverse pathogenesis of these two diseases.^44^ Notably, traditional risk factors for AS such as diabetes have shown unexpectedly divergent associations with AAA.^45^ The role of dyslipidaemia in AAA is particularly unclear. Large-scale genome-wide association studies have identified numerous independent lipid metabolism-related genetic risk loci in AS and AAA.^46–48^ Several studies, including ours, have also revealed opposing effects of key regulators of lipid metabolism (e.g. LXRα and PLA2G10) on AS and AAA.^16,36,49,50^ The present study underscores the distinct lipidomic metabolic features and associated pathogenic mechanisms underlying AS vs AAA. Specifically, neutral lipids, including triglycerides and cholesterol esters, were predominantly upregulated in AS, while phosphoglycerides were more significantly elevated in AAA animal models. These specific lipid metabolism features were further validated in human cohorts including hospital-based internal cohort and the UK Biobank cohort. Collectively, these results demonstrated the disease-specific associations of the identified differential lipid traits with AS and AAA, highlighting the distinct roles of lipid metabolism in the pathogenesis of these two diseases.

Through integrated transcriptomic and metabolomic analyses, we identified FA metabolism as the most significantly altered lipid-related pathway between AAA and AS. FAs are essential for energy storage, membrane synthesis, and production of signalling molecules.^51^ However, the role of FA metabolism in AS vs AAA has never been investigated.^52^ Our findings provide the first evidence that the FA metabolic pathway is differentially regulated in these two diseases, with significant downregulation observed in AS but not in AAA. Further analysis revealed that genes downregulated in AS were mainly enriched in FAO, the principal pathway for FA degradation. Aberrant FAO can result in excess lipid accumulation, contributing to various pathologies (e.g. renal injury and hepatic steatosis).^53,54^ Protein–protein interaction network analyses further identified *Acadm*, a key FAO enzyme, as the top-ranked node among FAO-related enzymes. Notably, defects in *Acadm* cause medium-chain acyl-CoA dehydrogenase deficiency (MCADD), a common hereditary metabolic disorder characterized by impaired FAO,^55^ underscoring its critical role in maintaining metabolic homeostasis. In our study, we observed pronounced suppression of *Acadm* expression and accumulation of its metabolic marker, medium-chain acylcarnitines, in AS but not in AAA, suggesting that disrupted FAO is specifically involved in atherogenesis. Mechanistically, *Acadm* overexpression reduced lipotoxic stress, contributing to the restored mitochondrial dysfunction and attenuated AS progression. Consistently, UK Biobank whole-exome sequencing data analysis revealed that loss-of-function mutations in *ACADM* were associated with increased carotid plaque burden, providing preliminary human genetic evidence for the role of *ACADM* in AS. Collectively, these results demonstrated that dysregulation of FA metabolism (especially the *Acadm*-mediated fatty acid β-oxidation) is the most significantly altered lipid-related pathway contributing to the lipid metabolic differences between these two vascular diseases.

Dyslipidaemia is characterized by an imbalance among lipid uptake, synthesis, and consumption. This lipid metabolic dysregulation leads to the intracellular accumulation of toxic lipid intermediates, ultimately causing cellular dysfunction and death.^56^ Lipid droplets are the primary organelles responsible for lipid storage. Under physiologic situations, LDs play a pivotal role in wrapping and sequestering excess lipids, primarily surplus free FAs, in less harmful esterified forms (such as triglycerides and cholesterol esters) within their neutral lipid core, thereby mitigating cellular lipotoxicity.^31^ Furthermore, LDs can be degraded, with triglycerides hydrolysed into free FAs for β-oxidation to generate energy.^31,57^ However, excessive accumulation of LDs is implicated in numerous pathological conditions, such as obesity,^58^ neurodegeneration,^59^ cancer,^60^ and particularly cardiovascular disease.^61,62^ Based on our recently developed non-protein-degrading strategy (ATTEC),^33^ we designed LD·ATTECs,^34^ which directly targeting LD degradation and can be utilized as a potent agent to investigate the role of LD accumulation in AS and AAA. We found that although LD·ATTEC effectively lowered LDs in both AS and AAA mice, it significantly attenuated atherosclerotic lesion formation in AS mice with little effects on aneurysm formation, suggesting a disease-specific role for LDs. Moreover, LD·ATTEC upregulated the expression of *Acadm* and restored the FAO pathway. We have previously demonstrated that LD·ATTEC mitigates age-related macular degeneration by degrading LDs.^63^ These findings provide convincing evidence for the distinct pathomechanisms of LD accumulation and FA metabolism in AAA and AS, highlighting the potential of LD-degrading strategy (i.e. LD·ATTEC and its optimized derivatives) for future applications.

The development of the AS and AAA is insidious, making early detection challenging before the onset of overt clinical symptoms, while traditional diagnostic methods exhibit limited sensitivity.^64,65^ Lipidomics provides a direct link to disease phenotypes, enabling the identification of potential biomarkers for clinical early diagnosis. In this study, we combined lipidomics with machine learning algorithms to develop predictive models for identifying and distinguishing CAD and AAA (*Figure 8E*). The AUC of the logistic regression model for CAD vs AAA was 0.968 (95% CI 0.946–0.990) in the training set and 0.841 (95% CI 0.747–0.935) in the validation set. Three additional classifiers, including RF, SVM, and XGBoost, also demonstrated robust performance in the validation set, with an average accuracy of 0.76, sensitivity of 0.80, specificity of 0.74, precision of 0.74, F1 score of 0.77, and AUC of 0.85 (Supplementary data online, *Table S8*). Therefore, lipid metabolite-based classifiers exhibit excellent discriminatory power, facilitating the accurate diagnosis and differential diagnosis of AS and AAA. Our machine learning–based prediction framework has been made publicly available on the online website for all interested researchers to explore (https://pu-lab.sjtu.edu.cn/shiny/lipidomics/).

### Study limitations

Several limitations should be considered. First, residual and unmeasured confounding cannot be entirely excluded despite adjustment for a broad range of demographic, lifestyle, clinical, and biological covariates, owing to imperfect measurement of confounders as well as the presence of factors that were unknown, unavailable, or not included in the models. Some confounders (e.g. smoking status) were self-reported and may be subject to measurement error or misclassification, which could bias effect estimates. In addition, hazard ratios in this study should be interpreted cautiously, as they are conditional on individuals who remain event-free and may vary with changes in the risk set over time, rather than reflecting a constant causal effect.^66^ Finally, although current findings support differential roles of ACADM in AS and AAA, the animal models used in this study are limited in their ability to fully recapitulate the complex pathophysiology of AS and AAA development in humans.

## Clinical implications

Our findings define previously unrecognized disease-specific lipid metabolic signatures in the pathogenesis of AS vs AAA in both preclinical animals and clinical patients, supporting that AS and AAA should be recognized as distinct diseases in terms of lipid metabolism. We identified fatty acid metabolism, particularly the *Acadm*-mediated FAO pathway, as the most significantly altered lipid metabolic pathway contributing to the lipid metabolic differences between AS and AAA, and further provided preliminary human genetic evidence supporting our findings on the critical role of *Acadm* in preclinical AS models. Restoration of FAO using our recently developed LD·ATTEC (PCT/CN2021/089453) significantly restored FAO and alleviated AS, providing a potential LD-degrading strategy for AS. Finally, we developed a machine learning predictive model based on lipid metabolic characteristics to discriminate between AS and AAA, establishing a foundation for the development of more effective, targeted therapeutic strategies. Future studies are warranted to further confirm our findings in large-scale population cohorts.

## Supplementary Material

ehag372_Supplementary_Data

## Declarations

### Data Availability

All raw data supporting the findings of the study are available upon the requests from the corresponding authors. Detailed description of materials and methods can be found in the Supplementary data. We also provided a web portal for easy data exploration (https://pu-lab.sjtu.edu.cn/shiny/lipidomics/).
