# Supplementary material for: ACADM-mediated fatty acid β-oxidation pathway in atherosclerosis and abdominal aortic aneurysm
Source: Eur Heart J. 2026 Jun 5;47(40):6051–72. doi: 10.1093/eurheartj/ehag372 (PMC13696078; doi:10.1093/eurheartj/ehag372)
Supplement: Supplementary file 1 [file ehag372_supplementary_data.docx]

**SUPPLEMENTAL MATERIAL**

**ACADM-mediated fatty acid β-oxidation pathway in atherosclerosis and abdominal aortic aneurysm**

Hengyuan Zhang^a※^, Lingyue Sun^a※^, Tingfeng Xu^b※^, Xiaowen Hou^a,c※^, Fangyuan Zhong^a^, Fanchao Shi^a^, Yifan Chen^a^, Yinan Li^c^, Yuli Wang^c^, Yuyan Lyu^a^, Wei Zhang^a^, Yuhua Fu^d^, Yichao Zhao^a^, Ancai Yuan^a^, Lei Chen^a^, Yong Ji^f,g^, Minxian Wang^b^, Boxun Lu^e^, Jun Pu ^a^*

^a^ Department of Cardiology, State Key Laboratory for Systems Medicine, Shanghai Cancer Institute, Renji Hospital, School of Medicine, Shanghai Jiao Tong University, Shanghai, China; ^b^ China National Center for Bioinformation, Beijing Institute of Genomics, Chinese Academy of Sciences, University of Chinese Academy of Sciences, Beijing, China; ^c^ Central laboratory, Ningbo Hangzhou Bay Hospital(Ningbo Branch of Renji Hospital, Shanghai Jiao Tong University School of Medicine, Shanghai), Ningbo, China; ^d^ Department of Vascular Surgery, Renji Hospital, School of Medicine, Shanghai Jiao Tong University, Shanghai, China; ^e^ State Key Laboratory of Medical Neurobiology and MOE Frontiers Center for Brain Science, School of Life Sciences, Fudan University, Shanghai, China; ^f^ Key Laboratory of Drug Targets and Translational Medicine for Cardio-cerebrovascular Diseases, Nanjing Medical University, Nanjing, China. ^g^ State Key Laboratory of Frigid Zone Cardiovascular Diseases (SKLFZCD), Harbin Medical University, Heilongjiang, China.

※These authors contributed equally to this work.

*Address for correspondence:

Jun Pu, MD, Ph.D., FACC, FESC

Department of Cardiology, State Key Laboratory for Systems Medicine for Cancer, Shanghai Cancer Institute, Renji Hospital, School of Medicine, Shanghai Jiao Tong University, Shanghai, China. E-mail: [pujun310@hotmail.com](mailto:pujun310@hotmail.com).

**Supplemental Methods**

***Myeloid Cell-specific Acadm Overexpression Mouse Construction***

Myeloid cell-specific *Acadm* overexpression mice (*Acadm^flox/flox^Lysm-Cre*, *Acadm^Tg^*) were generated by breeding *Acadm*-floxed mice with Lysm-Cre [B6.129P2-*Lyz2^tm1(cre)Ifo^/J*, #004781] mice from Jackson Laboratory. Then, *Acadm^Tg^* mice were crossed with *ApoE^-/-^* mice to establish *ApoE^-/-^/ Acadm^Tg^* mice. *ApoE^-/-^/Acadm^flox/flox^* littermates were used as controls.

***Animal Model of Rupture-Prone Vulnerable Plaques***

For the rupture-prone vulnerable plaque model, following our previously published protocol,^1,2^ the mice underwent ligation of the left internal and external carotid arteries, along with constriction of the left renal artery using a 0.12-mm pin gauge to maintain renal perfusion. The mice were then fed an HFD (40% kcal fat and 1.25% cholesterol) for 8 weeks.

***In vivo LD∙ATTEC Treatment***

To confirm the effects of LD∙ATTEC, wire injury-induced AS and Ang II-induced AAA mice were randomly divided into two groups: (1) vehicle group,1% DMSO (D2650, Sigma-Aldrich, USA) + 39% PEG300 (S6704, Selleck, China) + 5% Tween-80 (S6702, Selleck, China) + 55% H_2_O; (2) LD∙ATTEC group, LD∙ATTEC in DMSO vehicle. The LD∙ATTEC compound (ChemPartner, China) was administered for 4 weeks via a daily subcutaneous injection after the surgery (30 mg/kg/d, 100 μL per injection). LD∙ATTEC concentration was determined on the basis of our previous studies and pre-experimental results.^3^ In the rupture-prone vulnerable plaque mouse group, LD∙ATTEC was administered daily via intraperitoneal injection starting 4 weeks post-surgery, and the treatment continued for 4 weeks. At the study endpoints, plasma and whole aorta samples were collected. TC (A111-1-1; Nanjing Jiancheng, China), LDL-C (A113-1-1; Nanjing Jiancheng, China), TG (A110-1-1; Nanjing Jiancheng, China), FFA ([A042-2-1](http://www.njjcbio.com/products.asp?id=2594); Nanjing Jiancheng, China), acetyl-CoA (AKFA019M; Boxbio, China), and coenzymeA (E-BC-K907-M; Elabscience, China) levels were measured with the kits according to the manufacturer's instruction.

***Human Arterial Tissue Samples***

Atherosclerotic artery specimens were obtained from patients undergoing amputation due to lower extremity arteriosclerosis obliterans. Healthy control artery specimens were obtained from patients who underwent amputation for distal limb gangrene secondary to acute lower extremity arterial embolism caused by thrombus detachment from atrial fibrillation. Human AAA tissues were harvested from patients undergoing surgical repair, and healthy control samples obtained from abdominal aorta of brain-dead organ donors.

***Metabolomics and Proteomics Analysis in the UK Biobank***

The metabolomics profiling in the UK Biobank participants was generated using a high-throughput NMR-based metabolic biomarker profiling platform developed by Nightingale Health Ltd, as described previously.^4^ Metabolic biomarkers were measured from randomly selected EDTA plasma samples from ~ 280,000 UK Biobank participants. A total of 251 metabolic biomarkers (170 original measures and 81 percentage ratios) were quantified for each sample, including lipids, fatty acids, amino acids, ketone bodies, and other low-molecular-weight metabolic biomarkers (e.g. gluconeogenesis-related metabolites), as well as lipoprotein subclass distribution, particle size and composition, between June 2019 and April 2020 (Phase 1) and April 2020 and June 2022 (Phase 2) using eight spectrometers at Nightingale Health, based in Finland.

Olink proteomic profiling was conducted on blood plasma samples collected from 53,021 UKB participants using the Olink Explore 3072 platform. 2,922 unique proteins were measured across four panels containing cardiometabolic, inflammation, neurology, and oncology proteins. Details on sample selection, in addition to processing and quality control information for the Olink assay, are provided in previous publications.^5^

***Gene-based analysis of ACADM variants***

Gene-based burden analysis was performed using whole-exome sequencing data from the UK Biobank. High-confidence predicted loss-of-function (pLoF) variants were defined as variants determined by 1) LoFHC (LoFTEE high confidence) and 2) missense variants annotated by Ensembl Variant Effect Predictor (VEP) tool, with a minor allele frequency (MAF) < 0.001.^6^ Carotid plaque burden was assessed by carotid intima–media thickness (cIMT) measured using a CardioHealth Station ultrasound system (Panasonic Healthcare Corporation of America; Newark, NJ). Aggregated *ACADM* pLoF variants were tested at the gene level for association with cIMT using linear regression. Models were adjusted for age, sex, the first 10 genetic principal components, genotype array, and assessment center to control for population structure and technical heterogeneity in the UK Biobank data. Model assumptions, including residual normality, homoscedasticity, and linearity of continuous predictors, were assessed. Two-sided *P* values are reported, with effect sizes expressed as β coefficients (µm change in cIMT).

***Histology and Morphometric Analysis***

Tissues were harvested, fixed with 4% paraformaldehyde (PFA), embedded in paraffin, and serially sectioned. After the serial 6–8 μm sections were cut, the paraffin-embedded abdominal aorta sections were stained with hematoxylin and eosin for general morphology, Masson's trichrome for fibrosis detection and Elastin van Gieson (EVG) for elastin degradation. Oil Red O staining was performed on frozen sections. Briefly, tissues were snap-frozen in the optimal cutting temperature compound (4583; Tissue-TEK, USA) after collection. Frozen sections of 8 μm thickness were immersed in the working solution of Oil Red O for 30 min and washed with deionized water. Lesion sizes were determined by averaging three sections in each mouse. Lipid deposition and collagen content were detected using Oil Red O and Masson's trichrome staining, respectively, and measurements were performed using ImageJ software (Version 1.8.0, USA). In EVG staining, the degree of elastin degradation was graded with a semiquantitative method according to the 4-grade scale as previously described^7^: Grade 1: No Degradation; Grade 2: Mild Degradation; Grade 3: Severe Degradation; Grade 4: Aortic Rupture.

For AS quantification in whole aortas, each aorta was incubated with 4% PFA for at least 24 h at 4 °C and pinned. Atherosclerotic plaques were stained with 0.5% Oil Red O. The thoracic aorta and aortic arch were then cut open longitudinally and spread flat with the intimal side up for computer-assisted planimetric analysis. Atherosclerotic lesion size measurements were performed by blinded investigators using ImageJ software (Version 1.8.0, USA).

***Blood Pressure Measurements***

Blood pressure was measured by a noninvasive tail-cuff BP-2000 Blood Pressure Analysis System (Visitech Systems, USA). Mice were acclimated for at least 15 minutes prior to initiating measurements by placement in the restrainer on the warmed platform (37°C). Systolic blood pressure values were derived from an average of five measurements per animal.

***Vascular Ultrasound Imaging***

MUI was performed using a Vevo770 system with a 55-MHz transducer (VisualSonics Inc., Ontario, Canada). Under continuous inhalation anesthesia with isoflurane, mice were positioned headfirst in the scanner. MUI images were acquired in B-mode according to the manufacturer's instructions. Three measurements of abdominal aortic diameter along both the short and long axes were taken by a blinded investigator to determine maximum aortic diameters.

***Isolation and Stimulation of Macrophages***

BMDMs were isolated from the femurs and tibias as described previously.^8^ The mice were euthanized with an inhalation overdose of isoflurane (3%) and the bones were isolated, cut open with sterile scissors, and flushed with ice-cold sterile PBS using a syringe with a 27 G needle. The cells were filtered through a 40-μm cell strainer, centrifuged at 150 × *g* for 5 min, and resuspended in a culture medium supplemented with 30 ng/mL recombinant M-CSF (416-ML-05; R&D, USA). After 7 days of differentiation, macrophages were used for the stimulation experiments. Mature macrophages were treated with 100 μg/mL oxidized low-density lipoprotein (ox-LDL, MP6009, Maokang biotechnology, China) for 24 h, followed by treatment with vehicle or LD∙ATTEC (50 μM) for an additional 24h and then harvested for further analysis.

Overexpression lentiviruses for *Acadm*, as well as control lentiviruses, were synthesized by Genomeditech Co. Ltd (Shanghai, China). BMDMs were transduced with lentiviral in diluted media at a multiplicity of transduction (MOI) of 100 for 48h. Positive cells were selected after 48h of puromycin treatment for the following experiments, and the transfection efficiency was analyzed by Western blot.

Chloroquine (CQ,50μΜ; HY-17589A, MedChemExpress, China) and *Atg5* siRNA served to block the autophagy. Transfection of siRNA was performed using Lipofectamine 3000 reagent (L3000015; Thermo Fisher Scientific, USA) for 24h, according to the manufacturer's instructions.

***Immunofluorescence Staining***

Immunofluorescence staining was performed on paraformaldehyde-fixed and frozen sections. After blocking with 5% BSA for 1 h, sections were incubated with primary antibodies against LC3 II (1:1,000, CY5770, Abways, China) overnight at 4 °C, followed by incubation with an Alexa Fluor 555 goat anti-Rabbit secondary antibody (1:200, A-21428, Thermo Fisher Scientific, USA) for 1 h at 37 °C. The cryosections were then washed with 1× PBS and incubated for 30 min with BODIPY 493/503 (1:5,000 from a 1 mg/mL stock solution in DMSO, D3922, Thermo Fisher Scientific, USA). DAPI (1:1000; C1002, Beyotime Biotechnology, China) was used to identify the nuclei. The stained sections were viewed under a fluorescence microscope (DMI8, Leica, Germany).

***Real-time Quantitative PCR***

Total RNA was isolated using TRIzol (15596-026, Invitrogen, USA) and then used for cDNA synthesis using a PrimeScript RT reagent kit (RR047A, Takara, Japan). Quantitative RT-PCR was performed using a LightCycler® 480 System (Roche, Germany) and TB Green Premix Ex Taq™ (RR820A, Takara, Japan). *GAPDH* was used as the housekeeping genes. PCR primer sequences were as follows: *Acadm* (mouse) forward primer GAACTAAACATGGGCCAGCG and reverse primer AGCACCCATTGCGATCTTGA; *Gapdh* (mouse) forward primer AGGTCGGTGTGAACGGATTTG and reverse primer TGTAGACCATGTAGTTGAGGTCA. *Cpt2* (mouse) forward primer CAGCACAGCATCGTACCCA and reverse primer TCCCAATGCCGTTCTCAAAAT; *Acadl* (mouse) forward primer TCTTTTCCTCGGAGCATGACA and reverse primer GACCTCTCTACTCACTTCTCCAG; *Ndufab1* (mouse) forward primer CTGAGGGAATCCGGAGGAGA and reverse primer GTCTAACGTCAGTGGGGGTG; *ACADM* (human) forward primer GCCAGCGATGTTCAGATACTAGAGG and reverse primer CCAACAGCACCAGCAGCTACTAC; *GAPDH* (human) forward primer ACAACTTTGGTATCGTGGAAGG and reverse primer GCCATCACGCCACAGTTTC.

***Western Blot Analysis***

Proteins were extracted from the abdominal aortas of the mice and separated on 10% SDS-PAGE gels, followed by transfer to a nitrocellulose membrane. Then, the membrane was blocked in 5% non-fat dry milk in TBST for 1h and incubated overnight at 4°C with primary antibodies against ACADM (1:1000, ab92461, Abcam, USA) and GAPDH (1:5000, 2118T, Cell Signaling Technology, USA). After incubation with the horseradish peroxidase-conjugated anti-rabbit IgG (1:10000, 111-035-003, Jackson ImmunoResearch Laboratories, USA), the protein bands were visualized by enhanced chemiluminescence (WB012, Sharebio, China) using the BIO-RAD protein analysis system (Chemidoc, BIO-RAD, USA).

***Flow Cytometry Analysis***

Flow cytometry was performed using standard procedures.^9^ Briefly, 5×10^5^–10^8^ cells were harvested and labeled with 0.2 μg/mL BODIPY 493/503, incubated at 37 °C for 15 min, and washed with PBS. Data acquisition was performed with an LSRFortessa X-20 (BD Biosciences, USA) and analyzed using FlowJo software (Version 10.4, FlowJo LLC, USA).

***Measurement of the Oxygen Consumption Rate (OCR)***

Oxygen consumption was assessed using a Seahorse XF96 analyzer (Agilent, USA) following the manufacturer’s protocol. In brief, BMDMs (10,000–20,000 cells in 80 µL per well) were seeded into XF96-well microplates (102601-100; Agilent, USA) and subsequently subjected to the indicated treatments. Wells without cells were used as the background control. The accompanying sensor cartridge was hydrated and calibrated overnight in a CO₂-free incubator at 37 °C. Prior to the assay, culture medium was replaced with assay medium [XF DMEM base medium (pH 7.4) supplemented with 1 mmol/L pyruvate, 2 mmol/L glutamine, and 10 mmol/L glucose], and cells were incubated for 1 h in a CO₂-free incubator at 37°C. Compounds were loaded into injection ports to achieve final concentrations of 1.5 µmol/L oligomycin (first injection), 2.0 µmol/L FCCP (second injection), and 0.5 µmol/L rotenone/antimycin A (third injection), using the XF Cell Mito Stress Test Kit (103015-100; Agilent, USA). After the measurement, culture supernatants were removed, and cellular protein was extracted. OCR values were normalized to the total protein content in each well.

***Measurements of Mitochondrial Membrane Potential and Mitochondrial ROS***

Mitochondrial membrane potential was assessed in BMDMs using MitoTracker Green FM (HY-135056; MedChemExpress, China) and the JC-1 Mitochondrial Membrane Potential Assay Kit (MA0338; MeilunBio, China). Mitochondrial ROS levels were measured with MitoSOX Red (HY-D1055; MedChemExpress, China). Staining was performed according to the manufacturers’ instructions. Nuclei were subsequently counterstained with DAPI, and cells were imaged with a laser scanning confocal microscope.

***Absolute Quantitative Lipidomics***

The experiments and data analysis were supported by Shanghai BIOTREE Biotechnology. Lipids were extracted and normalized to equal wet weights of mouse aortic tissue and equal volumes of human plasma for liquid chromatography/mass spectrometry (LC–MS/MS). A quality control (QC) sample was prepared by mixing equal aliquots of the supernatant from each sample. UHPLC separation was performed using an ACQUITY Premier Series UHPLC System (Waters Corporation, USA). A SCIEX Triple Quad™ 6500+ (SCIEX, USA) mass spectrometer was applied for assay development. The absolute content of individual lipids corresponding to the IS was calculated based on the peak area and actual concentration of the lipid class internal standard. Lipid metabolites were classified according to LIPID MAPS consortium,^10^ including fatty acyls, glycerolipids, phosphoglycerides, sphingolipids, sterol lipids, saccharolipids, prenol lipids, and polyketides. Due to limitations of the detection method, only fatty acyls, glycerolipids, phosphoglycerides, sphingolipids and sterol lipids were analyzed. The specific classification of detected lipid metabolites is available in Supplemental Table S3.

***Acylcarnitine Profiling***

Acylcarnitines were quantified by LC–MS/MS. Tissue samples (~50 mg) were mixed with 75% methanol (50 mg/mL), vortexed for 1 h, and centrifuged at 12 000 rpm for 10 min at 4 °C; plasma (50 µL) was extracted with 150 µL methanol containing isotope-labeled internal standards (C0-D3 2000 ng/mL, C2-D3 1000 ng/mL, C4-D3 100 ng/mL, C8-D3 100 ng/mL, C16-D3 100 ng/mL). Supernatants were transferred to autosampler vials for analysis. Chromatographic separation was performed on a Waters ACQUITY UPLC I-CLASS system using an HSS T3 column (1.7 µm, 2.1 × 150 mm) at 50 °C with a mobile phase of water/0.1% formic acid (A) and acetonitrile:isopropanol (88:12, B) at 0.26 mL/min. Detection was achieved on a Waters XEVO TQ-XS triple quadrupole mass spectrometer in positive electrospray mode (capillary 3.0 kV, source 150 °C, desolvation 500 °C, desolvation gas 950 L/h, cone gas 10 L/h). Quantification was performed using TargetLynx software by integrating peak areas and normalizing to isotope-labeled internal standards.

***UHPLC-QE-MS Untargeted-metabolomics***

The experiments and data analysis were supported by Shanghai BIOTREE Biotechnology. Metabolites were extracted from the samples for analysis. A QC sample was prepared by mixing an equal aliquot of the supernatants from all samples, and LC-MS/MS analyses were performed using a UHPLC system with a UPLC BEH Amide column (2.1×100 mm, 1.7 μm; Waters Corporation, USA). Raw data were processed for peak detection, extraction, alignment, and integration. The MS2 database was used for metabolite annotation.

***RNA Sequencing***

Total RNA from the aortas was extracted using TRIzol reagent. RNA purity and quantification were evaluated using the NanoDrop 2000 spectrophotometer (Thermo Scientific, USA). RNA integrity was assessed using the Agilent 2100 Bioanalyzer (Agilent Technologies, Santa Clara, CA, USA). Then the libraries were constructed using VAHTS Universal V6 RNA-seq Library Prep Kit (Vazyme, China). Sequencing was performed using a Solexa high-throughput sequencing service (Oebiotech, Shanghai, China). Differential expression analyses were performed using the DESeq R package (DESeq version: 1.30.0, R version: 4.3.2). In this project, fold change ≥2 (|log_2_FC| ≥1) and *P*-value <0.05 were used as the threshold criteria for screening differentially expressed genes (|log*_2_*FC|≥1 & *P*<0.05). Based on the hypergeometric distribution, GO analysis of the differentially expressed genes (DEGs) was performed to screen for significantly enriched terms using R (v 4.3.2).

***Machine Learning***

Machine learning was employed to identify the most predictive lipid metabolites. Raw lipidomic data were normalized by sum and auto-scaled to address large variations and non-normal distributions. To mitigate overfitting and ensure model robustness and generalizability, the study cohort was randomly partitioned into a training set (70%) for feature selection and model development, and an independent validation set (30%) for performance evaluation, a widely adopted split ratio selected to ensure sufficient statistical information for model training while preserving independent validation precision.^11,12^ Additionally, 10-fold cross-validation was employed within the training set to evaluate model performance. The average prediction performance metrics are presented in Supplementary Table S6. Feature selection was performed strictly within the training cohort using a three-step strategy: (1) Student’s *t*-test (*P* < 0.05), (2) LASSO regression with the regularization parameter (λ) selected via 10-fold cross-validation using the minimum mean cross-validated error criterion (λ_min), and (3) partial least squares-discriminant analysis (PLS-DA) retaining features with variable importance in projection (VIP) > 1. Subsequently, the refined feature set was incorporated into a multivariable logistic regression model adjusted for clinical covariates (age, sex, and body mass index), and model performance was evaluated exclusively on the held-out validation set to provide an unbiased estimate of predictive accuracy. We evaluated four traditional machine learning methods, including logistic regression, random forest (RF), support vector machines (SVM), and extreme gradient boosting (XGBoost). Additionally, a Super Learner ensemble model was constructed as described by Van der Laan et al.^13^ The base learner library included SL.glm (logistic regression), SL.glmnet (LASSO), SL.ranger (random forest), SL.xgboost (XGBoost), and SL.mean (sample mean). The Super Learner was fitted using 10-fold cross-validation with non-negative log-likelihood optimization. Ensemble weights were constrained to be non-negative and sum to one. Model performance was assessed using various metrics: accuracy, sensitivity, specificity, precision (positive predictive value), F1 score (harmonic mean of sensitivity and precision), and area under the curve (AUC). Pairwise DeLong tests were used to compare AUC values between different models (logistic regression, RF, SVM, XGBoost, and Super Learner) for each group comparison (CAD *vs.* AAA, AAA *vs.* HC, CAD *vs.* HC). A two-sided *P* < 0.05 was considered statistically significant (Supplemental Table S7).

***Supplemental Statistical Analysis***

Blinding was used in the study. According to our randomization and blinding strategy, animals were grouped by Dr. Zhong, and the surgery was operated by Dr. Zhang. The color Doppler ultrasound was carried out by Dr. Sun. Histology analysis was performed by Dr. Hou, assisted by Dr. Xu. The researchers except Dr. Zhong were unaware of the treatment group assignment or genotype.

**Supplemental Results**

**Differential associations of baseline lipid traits with risk of CAD and AAA in UK** **Biobank cohort**

To explore longitudinal associations between lipid traits and the risks of AS and AAA, we leveraged data from the UK Biobank. Our analysis included 479,256 adults without CAD or AAA at baseline, with a median age of 57 years, 55.7% of whom were female. Supplemental Table S4 presents the baseline characteristics of participants according to disease status. During a median follow-up of 14.8 years (IQR: 14.1,15.5 years), 22,675 incident CAD cases and 2,159 incident AAA cases were identified. Considering the differences in the baseline characteristics between the groups, we employed Cox proportional hazards models adjusted for baseline characteristics (e.g., gender, age at enrollment, BMI, smoking status, medication use, and assessment center) to evaluate the associations of baseline metabolic traits with incident CAD and AAA. Metabolomic analyses identified 160 metabolites associated with incident CAD, and 112 metabolites associated with incident AAA (Fig. S3A and S3B). The number and composition of metabolites significantly associated with incident CAD and incident AAA varied substantially (Fig. S3C). Metabolic analysis provides simultaneous quantification of routine lipids, lipoprotein subclass, and low-molecular weight metabolites,^10^ thus, we first analyzed the association between the levels of routine lipid metabolites^10^ and the risk of CAD and AAA (Fig. S3D). Total triglycerides exhibited the strongest positive association (HR 1.19, 95% CI 1.17–1.21) with CAD risk, followed by LDL cholesterol (HR 1.14, 95% CI 1.12–1.17), total fatty acids (HR 1.14, 95% CI 1.12–1.16), total free cholesterol (HR 1.14, 95% CI 1.12–1.16), and total cholesterol (HR 1.09, 95% CI 1.07–1.11). In contrast, phosphatidylcholines showed the strongest negative association (HR 0.86, 95% CI 0.81–0.91) with AAA risk, followed by phosphoglycerides (HR 0.89, 95% CI 0.83–0.94) and total cholesterol (HR 0.94, 95% CI 0.88–0.99).

To minimize potential influences of baseline characteristic differences on our findings, we further conducted PSM analyses. After 1:3 matching, three matched CAD participants and three matched controls were randomly selected for each AAA participant. A total of 1,209 AAA participants, 3,573 matched CAD participants, and 3,624 matched controls were included for analysis. After PSM, no significant difference in baseline characteristics were observed across the control, CAD, and AAA groups (Supplemental Table S5). The differences in metabolites between CAD and AAA remained significant, consistent with our above findings in both lipid classes and correlation pattern in the total population (Fig. S4A and S4B). Notably, results accounting for competing mortality risk using Fine and Gray competing risk models were broadly consistent with the main analyses from the Cox models (Fig. S4C). Heatmap analyses of metabolites further highlighted distinct metabolomic profiles between CAD and AAA (Fig. S3E). We further evaluated the consistency and heterogeneity in metabolite associations for CAD and AAA. The results revealed differential associations routine lipid metabolites with incident CAD and AAA (Figure. S3F).

We further explored the associations between plasma lipid-related proteins and the incidence of CAD and AAA. Proteomics analysis identified 1,650 proteins associated with incident CAD and 464 associated with incident AAA (Fig. S3G and S3H). To focus on proteins linked to lipid metabolism, we retrieved relevant proteins from NCBI (https://www.ncbi.nlm.nih.gov/), UniProt (https://www.uniprot.org/), and Lipid Maps (https://lipidmaps.org/) database. Alterations in lipid-related proteins were more prominently observed in CAD (Fig. S3I). Enrichment analyses of intersecting proteins revealed significant enrichment in FA binding and FA β-oxidation pathways in CAD (Fig. S3J). In contrast, phospholipid binding emerged as the most significantly enriched lipid metabolism-related pathway in AAA (Fig. S3K). Collectively, these metabolic and proteomic data from the UK Biobank highlight the distinct roles of lipid metabolic disturbances in the risk of AS and AAA.

**LD∙ATTEC reduces LDs in macrophages in an autophagy-dependent manner**

The effects of LD-degrading strategies in vascular diseases have never been investigated. To investigate the mechanism through which LD∙ATTEC promotes LD degradation and reduces LD accumulation in macrophages, we isolated BMDMs from mice and treated them with LD∙ATTEC following LD induction with ox-LDL. Then we co-stained autophagosomes and LDs. After treatment with LD∙ATTEC, significant colocalization between autophagosomes and LDs was observed in ox-LDL-treated BMDMs, but not in the controls (Fig. S9A). This colocalization was also confirmed in mouse aortic tissues, where a marked increase in the colocalization of autophagosomes with LDs was observed after LD∙ATTEC treatment (Fig. S9B). Moreover, the reduction of LDs by LD∙ATTEC was largely inhibited by the autophagy inhibitor chloroquine (CQ) (Fig. S9C) or knockdown of the key autophagy gene *Atg5* (Fig. S9D and S9E), confirming its autophagy dependence. Collectively, these data suggest that LD∙ATTEC reduces LD accumulation by enhancing their degradation in macrophages via autophagy.

**Association between *ACADM* loss-of-function mutations and carotid plaque burden**

Building upon the experimental findings on the critical role of *Acadm* in AS animal models, we further evaluated the clinical relevance of *ACADM* loss-of-function mutations in human AS. We analyzed whole-exome sequencing data from the UK Biobank to explore whether impaired *ACADM* function, inferred from pLoF variants, was associated with carotid plaque burden (measured by cIMT) (Fig. S16 A). A total of 14 high-confidence pLoF variants of *ACADM* associated with AS were identified from UK Biobank whole-exome sequencing data. High-confidence pLoF variants were defined as variants determined by 1) LoFHC (LoFTEE high confidence) and 2) missense variants annotated by VEP tool, with a minor allele frequency (MAF) < 0.001^5^ (Supplemental Table S8). Carriers of these variants exhibited significantly greater cIMT compared with noncarriers (95% CI = 13.25–138.70; *P* = 0.0176), indicating that loss-of-function mutations in *ACADM* were associated with increased AS burden (Fig. S16B and S16C). These results provide complementary human genetic evidence supporting the protective role of ACADM observed in AS mouse models.

**References**

1. Jin SX, Shen LH, Nie P, Yuan W, Hu LH, Li DD, et al. Endogenous renovascular hypertension combined with low shear stress induces plaque rupture in apolipoprotein E-deficient mice. *Arterioscler Thromb Vasc Biol* 2012;**32**:2372-2379. doi: https://doi.org/10.1161/atvbaha.111.236158
2. Che X, Xiao Q, Song W, Zhang H, Sun B, Geng N, et al. Protective Functions of Liver X Receptor α in Established Vulnerable Plaques: Involvement of Regulating Endoplasmic Reticulum-Mediated Macrophage Apoptosis and Efferocytosis. *J Am Heart Assoc* 2021;**10**:e018455. doi: https://doi.org/10.1161/jaha.120.018455
3. Fu Y, Chen N, Wang Z, Luo S, Ding Y, Lu B. Degradation of lipid droplets by chimeric autophagy-tethering compounds. *Cell Res* 2021;**31**:965-979. doi: https://doi.org/10.1038/s41422-021-00532-7
4. Eldjarn GH, Ferkingstad E, Lund SH, Helgason H, Magnusson OT, Gunnarsdottir K, et al. Large-scale plasma proteomics comparisons through genetics and disease associations. *Nature* 2023;**622**:348-358. doi: https://doi.org/10.1038/s41586-023-06563-x
5. Carrasco-Zanini J, Pietzner M, Davitte J, Surendran P, Croteau-Chonka DC, Robins C, et al. Proteomic signatures improve risk prediction for common and rare diseases. *Nat Med* 2024. doi: https://doi.org/10.1038/s41591-024-03142-z
6. Backman JD, Li AH, Marcketta A, Sun D, Mbatchou J, Kessler MD, et al. Exome sequencing and analysis of 454,787 UK Biobank participants. *Nature* 2021;**599**:628-634. doi: https://doi.org/10.1038/s41586-021-04103-z
7. Sun LY, Lyu YY, Zhang HY, Shen Z, Lin GQ, Geng N, et al. Nuclear Receptor NR1D1 Regulates Abdominal Aortic Aneurysm Development by Targeting the Mitochondrial Tricarboxylic Acid Cycle Enzyme Aconitase-2. *Circulation* 2022;**146**:1591-1609. doi: https://doi.org/10.1161/circulationaha.121.057623
8. Zhang H, Geng N, Sun L, Che X, Xiao Q, Tao Z, et al. Nuclear Receptor Nur77 Protects Against Abdominal Aortic Aneurysm by Ameliorating Inflammation Via Suppressing LOX-1. *J Am Heart Assoc* 2021;**10**:e021707. doi: https://doi.org/10.1161/jaha.121.021707
9. Wu H, Han Y, Rodriguez Sillke Y, Deng H, Siddiqui S, Treese C, et al. Lipid droplet-dependent fatty acid metabolism controls the immune suppressive phenotype of tumor-associated macrophages. *EMBO Mol Med* 2019;**11**:e10698. doi: https://doi.org/10.15252/emmm.201910698
10. Fahy E, Subramaniam S, Murphy RC, Nishijima M, Raetz CR, Shimizu T, et al. Update of the LIPID MAPS comprehensive classification system for lipids. *J Lipid Res* 2009;**50 Suppl**:S9-14. doi: https://doi.org/10.1194/jlr.R800095-JLR200
11. Gill SK, Karwath A, Uh HW, Cardoso VR, Gu Z, Barsky A, et al. Artificial intelligence to enhance clinical value across the spectrum of cardiovascular healthcare. *Eur Heart J* 2023;**44**:713-725. doi: https://doi.org/10.1093/eurheartj/ehac758
12. Patel A, Göbel K, Ille S, Hinz F, Schoebe N, Bogumil H, et al. Prospective, multicenter validation of a platform for rapid molecular profiling of central nervous system tumors. *Nat Med* 2025;**31**:1567-1577. doi: https://doi.org/10.1038/s41591-025-03562-5
13. van der Laan MJ, Polley EC, Hubbard AE. Super learner. *Stat Appl Genet Mol Biol* 2007;**6**:Article25. doi: https://doi.org/10.2202/1544-6115.1309

**Supplemental Tables**

**Table S1. Clinical characteristics of subjects included in the lipidomic analysis in Renji hospital cohorts**

|  | **Controls** | **CAD** | **AAA** | ***P*** |
| --- | --- | --- | --- | --- |
| **Age, years** | 73.88 ± 8.09 | 72.06 ± 9.02 | 72.82 ± 9.23 | 0.269 |
| **Male sex** | 102 (84.30%) | 97 (80.17%) | 96 (79.34%) | 0.571 |
| **BMI, kg/m^2^** | 23.55 ± 3.02 | 24.46 ± 3.22 | 23.67 ± 3.29 | 0.055 |
| **RBC,** ×10^12^/L | 5.08 ± 0.44 | 5.14 ± 0.42 | 5.06 ± 0.42 | 0.332 |
| **MCV, fl** | 87.42 ± 2.89 | 87.18 ± 3.04 | 87.33 ± 3.47 | 0.833 |
| **Urea, mmol/L** | 7.60 ± 0.31 | 7.63 ± 0.27 | 7.6 1 ± 0.22 | 0.677 |
| **Creatinine, μmol/L** | 68.87(64.14, 77.15) | 67.83(63.89, 72.27) | 68.94(64.78, 72.26) | 0.594 |
| **ALT, U/L** | 39.23(37.18, 40.38) | 39.02(38.44, 39.91) | 38.61(37.2, 40.4) | 0.488 |
| **AST, U/L** | 26.72(25.84, 27.8) | 26.79(26.28, 27.28) | 27.2(25.49, 28.72) | 0.177 |
| **Blood lipid concentrations** |  |  |  |  |
| TG, mmol/L | 1.29 ± 0.80 | 1.56 ± 0.81 | 1.60 ± 0.93 | 0.007 |
| TC, mmol/L | 3.93 ± 0.80 | 4.77 ± 0.99 | 4.71 ± 0.93 | <0.001 |
| HDL-C, mmol/L | 1.13 ± 0.34 | 1.16 ± 0.33 | 1.26 ± 0.34 | 0.007 |
| LDL-C, mmol/L | 2.27 ± 0.71 | 2.90 ± 0.86 | 2.75 ± 0.79 | <0.001 |
| **Smoking status, *n* (%)** |  |  |  | <0.001 |
| Never | 90 (74.38%) | 52 (42.98%) | 40 (33.06%) |  |
| Previous or current | 31 (25.62%) | 69 (57.02%) | 81 (66.94%) |  |
| **Lipid-lowering drug, *n* (%)** | 0 (0.00%) | 60 (49.59%) | 54 (44.63%) | < 0.001 |
| **Pressure-lowering drug, *n* (%)** | 1 (0.83%) | 9 (7.44%) | 12 (9.92%) | 0.005 |

Continuous variables are presented as mean ± SD for approximately symmetric distributions and median (IQR) for skewed distributions; categorical variables are presented as n (%). CAD indicates coronary artery disease; AAA, abdominal aortic aneurysm; BMI, body mass index; TC, total cholesterol; TG, triglycerides; HDL-C, high-density lipoprotein-cholesterol; LDL-C, low-density lipoprotein-cholesterol; RBC, red blood cell; MCV, mean corpuscular volume; ALT, alanine aminotransferase; AST, aspartate aminotransferase.

**Table S2. Definitions for CAD and AAA in the UK Biobank**

| **Outcome** | **Data Code** |
| --- | --- |
| Coronary artery disease | 410,4109,411,4119,412,4129,4140,4148,4149,I21,I21.0,I21.1,I21.2,I21.3,I21.4,I21.9,I22,I22.0,I22.1,I22.8,I22.9,I23,I23.0,I23.1,I23.2,I23.3,I23.4,I23.5,I23.6,I23.8,I24.0,I24.1,I24.8,I24.9,I25.0,I25.1,I25.2,I25.5,I25.6,I25.8,I25.9,K49,K49.1,K49.2,K49.3,K49.4,K49.8,K49.9,K75,K75.1,K75.2,K75.3,K75.4,K75.8,K75.9,K50.1,K50.2,K50.4,K40,K40.1,K40.2,K40.3,K40.4,K40.8,K40.9,K41,K41.1,K41.2,K41.3,K41.4,K41.8,K41.9,K42,K42.1,K42.2,K42.3,K42.4,K42.8,K42.9,K43,K43.1,K43.2,K43.3,K43.4,K43.8,K43.9,K44,K44.1,K44.2,K44.8,K44.9,K45.1,K45.2,K45.3,K45.4,K45.5,K45.6,K45.8,K45.9,K46,K46.1,K46.2,K46.3,K46.4,K46.5,K46.8,K46.9,K47.1 |
| Abdominal aortic aneurysm | I71.3, I71.4, L18, L19, L25.4, L27, L28, L46.4, 1591 |

**Table S3. The list of lipid classification**

| **Lipid Class** | **Lipid Subclass** |
| --- | --- |
| Fatty acyls | Free Fatty Acids (FFA) |
| Glycerolipids | Monogalactosyldiacylglycerol (MGDG) |
|  | Digalactosyldiacylglycerol (DGDG) |
|  | Diglycerides (DAG) |
|  | Triglycerides (TAG) |
| Sterol lipids | Cholesterol Esters (CE) |
| Sphingolipids | Ceramides (CER) |
|  | Dihydroceramides (DCER) |
|  | Hexosylceramides (HCER) |
|  | Lactosylceramides (LCER) |
|  | Sphingomyelins (SM) |
| Phosphoglycerides | Lysophosphatidylcholines (LPC) |
|  | Lysophosphatidylethanolamines (LPE) |
|  | Lyso-phosphatidylglycerol (LPG) |
|  | Lyso-phosphatidylinositol (LPI) |
|  | Lyso-phosphatidylserine (LPS) |
|  | Phosphatidylcholines (PC) |
|  | Phosphatidylethanolamines (PE) |
|  | Phosphatidylglycerol (PG) |
|  | Phosphatidylinositol (PI) |
|  | Phosphatidylserine (PS) |
|  | Phosphatidylinositol phosphate (PIP) |
|  | Bis(monoacylglycero)phosphate (BMP) |

**Table S4. Baseline characteristics of the all participants included in the UK Biobank study**

| **Baseline characteristics** | **Overall**  ***n*=** **479,256** | **Participants with CAD**  **at follow-up**  ***n*=** **22,675** | **Participants with AAA**  **at follow-up**  ***n*=** **2,159** |
| --- | --- | --- | --- |
| **Age (years)** | 57.0 [50.0,63.0] | 61.0 [56.0,66.0] | 64.0 [60.0,67.0] |
| **Male sex, *n* (%)** | 212263 (44.3) | 15590 (68.8) | 1702 (78.8) |
| **BMI (kg/m^2^)** | 27.36 ± 4.78 | 28.64 ± 4.87 | 28.23 ± 4.42 |
| **Smoking status, *n* (%)** |  |  |  |
| Never | 266138 (55.5) | 10034 (44.3) | 527 (24.4) |
| Previous or current | 213118 (44.5) | 12641 (55.7) | 1632 (75.6) |
| **Lipid-lowering drug, *n* (%)** | 71726 (15.0) | 6771 (29.9) | 711 (32.9) |
| **Pressure-lowering drug, *n* (%)** | 1472 (0.3) | 153 (0.7) | 16 (0.7) |
| **Follow-up time, years** | 14.8 [14.1,15.5] | 14.6 [13.5,15.5] | 14.4 [13.1,15.4] |

Continuous variables are presented as mean ± SD for approximately symmetric distributions and median [IQR] for skewed distributions; categorical variables are presented as n (%).

Abbreviations: BMI, body mass index.

**Table S5. Baseline characteristics of the propensity score matched participants included in the UK Biobank study**

| **Baseline characteristics** | **Controls**  ***n*=** **3,627** | **Participants with CAD**  **at follow-up**  ***n*=** **3,627** | **Participants with AAA**  **at follow-up**  ***n*=** **1,209** | ***P*-Value** |
| --- | --- | --- | --- | --- |
| **Age (years)** | 64.0 [60.0,67.0] | 64.0 [60.0,67.0] | 64.0 [60.0,67.0] | 0. 436 |
| **Male sex, *n* (%)** | 2883 (79.5) | 2883 (79.5) | 957 (79.2) | 0.754 |
| **BMI (kg/m^2^)** | 28.26 ± 4.49 | 28.24 ± 4.51 | 28.26 ± 4.49 | 0.976 |
| **Smoking status, *n* (%)** |  |  |  | 0.311 |
| Never | 927 (25.6) | 873 (24.1) | 307 (25.4) |  |
| Previous or current | 2700 (74.4) | 2754 (75.9) | 902 (74.6) |  |
| **Lipid-lowering drug, *n* (%)** | 1169 (32.2) | 1197 (33.0) | 395 (32.7) | 0.781 |
| **Pressure-lowering drug, *n* (%)** | 14 (0.4) | 21 (0.6) | 7 (0.6) | 0.458 |
| **Follow-up time, years** | 14.7 [13.8,15.5] | 14.5 [12.9,15.4] | 14.5 [13.4,15.4] |  |

Continuous variables are presented as mean ± SD for approximately symmetric distributions and median [IQR] for skewed distributions; categorical variables are presented as n (%).

Abbreviations: BMI, body mass index.

**Table S6. DeLong test for AUC comparison**

| **Model** | **AUC (95% CI)** | **10-fold CV AUC (95% CI)** |
| --- | --- | --- |
| **CAD *vs.* AAA** |  |  |
| Logistic | 0.968 (0.946, 0.990) | 0.894 (0.843, 0.944) |
| RF | 1.000 (1.000, 1.000) | 0.880 (0.824, 0.935) |
| SVM | 0.928 (0.889, 0.967) | 0.872 (0.820, 0.924) |
| XGBoost | 1.000 (1.000, 1.000) | 0.870 (0.801, 0.939) |
| Super Learner | 0.998 (0.994, 1.000) | 0.913 (0.871, 0.954) |
| **CAD *vs.* HC** |  |  |
| Logistic | 0.974 (0.954, 0.994) | 0.924 (0.899, 0.950) |
| RF | 1.000 (1.000, 1.000) | 0.927 (0.904, 0.951) |
| SVM | 0.939 (0.903, 0.976) | 0.935 (0.901, 0.969) |
| XGBoost | 1.000 (1.000, 1.000) | 0.917 (0.878, 0.956) |
| Super Learner | 0.996 (0.992, 1.000) | 0.943 (0.910, 0.975) |
| **AAA *vs.* HC** |  |  |
| Logistic | 0.913 (0.870, 0.956) | 0.809 (0.702, 0.917) |
| RF | 1.000 (1.000, 1.000) | 0.801 (0.736, 0.867) |
| SVM | 0.940 (0.904, 0.976) | 0.770 (0.686, 0.855) |
| XGBoost | 1.000 (1.000, 1.000) | 0.823 (0.769, 0.878) |
| Super Learner | 1.000 (1.000, 1.000) | 0.816 (0.751, 0.880) |

Abbreviations: AAA, abdominal aortic aneurysm; CAD, coronary artery disease; HC, healthy control; RF, random forest; SVM, support vector machine; CV, cross-validation.

**Table S7. DeLong test for AUC comparison**

| **Model1** | **Model2** | **AUC1** | **AUC2** | **DeLong_*P*** |
| --- | --- | --- | --- | --- |
| **AAA *vs.* CAD** |  |  |  |  |
| Logistic | RF | 0.841 | 0.867 | 0.489 |
| Logistic | SVM | 0.841 | 0.830 | 0.717 |
| Logistic | XGBoost | 0.841 | 0.863 | 0.581 |
| Logistic | SuperLearner | 0.841 | 0.851 | 0.665 |
| RF | SVM | 0.867 | 0.830 | 0.281 |
| RF | XGBoost | 0.867 | 0.863 | 0.903 |
| RF | SuperLearner | 0.867 | 0.851 | 0.535 |
| SVM | XGBoost | 0.830 | 0.863 | 0.395 |
| SVM | SuperLearner | 0.830 | 0.851 | 0.439 |
| XGBoost | SuperLearner | 0.863 | 0.851 | 0.683 |
| **CAD *vs.* HC** |  |  |  |  |
| Logistic | RF | 0.832 | 0.844 | 0.587 |
| Logistic | SVM | 0.832 | 0.854 | 0.344 |
| Logistic | XGBoost | 0.832 | 0.815 | 0.640 |
| Logistic | SuperLearner | 0.832 | 0.837 | 0.725 |
| RF | SVM | 0.844 | 0.854 | 0.757 |
| RF | XGBoost | 0.844 | 0.815 | 0.171 |
| RF | SuperLearner | 0.844 | 0.837 | 0.606 |
| SVM | XGBoost | 0.854 | 0.815 | 0.168 |
| SVM | SuperLearner | 0.854 | 0.837 | 0.372 |
| XGBoost | SuperLearner | 0.815 | 0.837 | 0.311 |
| **AAA *vs.* HC** |  |  |  |  |
| Logistic | RF | 0.789 | 0.767 | 0.628 |
| Logistic | SVM | 0.789 | 0.764 | 0.153 |
| Logistic | XGBoost | 0.789 | 0.790 | 0.986 |
| Logistic | SuperLearner | 0.789 | 0.767 | 0.645 |
| RF | SVM | 0.767 | 0.764 | 0.315 |
| RF | XGBoost | 0.767 | 0.790 | 0.433 |
| RF | SuperLearner | 0.767 | 0.767 | 0.865 |
| SVM | XGBoost | 0.764 | 0.790 | 0.193 |
| SVM | SuperLearner | 0.764 | 0.767 | 0.325 |
| XGBoost | SuperLearner | 0.790 | 0.767 | 0.478 |

**Table S8. pLoF variants in *ACADM* identified from UK Biobank whole-exome sequencing.**

| **rsID** | **VariantID** | **Cases** |
| --- | --- | --- |
| rs1225471006 | 1:75761173:G:GCTAGAATGAGTTA | 1 |
| rs748110745 | 1:75761326:G:T | 3 |
| rs387906297 | 1:75761275:CAGTT:C | 1 |
| rs3841786 | 1:75762761:T:TA | 2 |
| rs1057516278 | 1:75761289:T:TG | 1 |
| rs866388216 | 1:75740111:G:A | 2 |
| rs875989873 | 1:75733559:TTTAA:T | 1 |
| rs1057516480 | 1:75732859:GT:G | 1 |
| rs786204642 | 1:75734848:ATGAC:A | 1 |
| rs1462279583 | 1:75749437:C:T | 1 |
| rs2100363479 | 1:75732866:T:TA | 1 |
| rs113887538 | 1:75728489:G:T | 1 |
| \ | 1:75745873:AT:A | 1 |
| rs769331400 | 1:75761371:G:A | 1 |

“rsID” indicates the dbSNP ID. “Variant ID” defined as the chromosome, position, reference allele and alternative allele. “Cases” indicates the number of variant carriers. Variants were characterized as LoFHC (LoFTEE high-confidence) and variants annotated by VEP with a minor allele frequency (MAF) < 0.001.

**Supplemental Figures**

**Supplemental Figure 1**

**Figure S1.** **Differential associations of plasma lipids with two vascular diseases (AS *vs.* AAA) in mice.**

**(A)** Correlation of aortic lesion area percentage with plasma levels of TC, LDL-C, and TAG in wire injury-induced AS (*n*=10-20 mice/group as indicated by the number of individual dots). The *P* values and correlation coefficients (ρ) were determined using Spearman's rank correlation analysis. **(B)** Correlation of aortic lesion area percentage with plasma levels of TC, LDL-C, and TAG in HFD-induced AS (*n*=10-20 mice/group as indicated by the number of individual dots). The *P* values and correlation coefficients (ρ) were determined using Spearman's rank correlation analysis. **(C)** Correlation of aortic diameters with plasma levels of TC, LDL-C, and TAG in Ang II-induced AAA (*n*=10–20 mice/group as indicated by the number of individual dots). The *P* values and correlation coefficients (ρ) were determined using Spearman's rank correlation analysis. **(D)** Correlation of aortic diameters with plasma levels of TC, LDL-C, and TAG in CaPO_4_-induced AAA (*n*=10-20 mice/group as indicated by the number of individual dots). Correlation coefficients were calculated using both Spearman’s rank correlation (ρ) and Pearson correlation (r), and corresponding 95% confidence intervals are reported. The linear regression line represents the ordinary least squares (OLS) fit, with the shaded area indicating the 95% confidence interval of the regression line. Abbreviations: AAA, abdominal aortic aneurysm; AS, atherosclerosis; TC, total cholesterol; LDL-C, low-density lipoprotein cholesterol; TG, triglycerides; HFD, high-fat diet.

**Supplemental Figure 2**

**Figure S2. Heatmap of differential lipid metabolites in AS and AAA mice.** Abbreviations: AAA, abdominal aortic aneurysm; AS, atherosclerosis; CE, cholesterol esters; DAG, diglycerides; FFA, free fatty acids; TAG, triglycerides.

**Supplemental Figure 3**

**Figure S3. Differential associations of baseline lipid traits and lipid metabolism-related proteins with risk of CAD and AAA** **in the UK Biobank cohort.**

**(A, B)** Associations of metabolites with the risk of CAD and AAA. Metabolites outside the red circle were significantly associated with the risk of CAD and AAA (FDR-adjusted *P* values < 0.05). **(C)** Stacked bar plots showing the number and composition of metabolites significantly associated with the risk of CAD and AAA. **(D)** Plot showing the associations of routine lipids with the risk of CAD and AAA. **(E)** Heat map showing differential associations pattern of metabolites with the risk of CAD and AAA from NMR-based metabolomics. **(F)** Consistency and heterogeneity of metabolites associations with the risk of CAD and AAA. The hazard ratio of each metabolite is given with 95% CIs in gray vertical and horizontal error bars. Metabolites with *P* ≤0.01 for heterogeneity between associations with CAD and AAA are marked by red dots. **(G, H)** Manhattan plots showing the differential associations pattern of proteins with the risk of CAD and AAA. Proteins above the horizontal dotted black line were significantly associated with the risk of CAD and AAA (FDR-adjusted *P* values < 0.05). **(I)** Venn diagram illustrating the overlap of lipid metabolism-related proteins significantly associated with the risk of CAD and AAA. **(J, K)** Enrichment analyses of lipid metabolism-related proteins associated with the risk of CAD and AAA, highlighting significant pathways identified through GO and Reactome. Abbreviations: AAA, abdominal aortic aneurysm; CAD, coronary artery disease; CE, cholesterol esters; GO, Gene Ontology; BP, biological process; CC, cellular component; MF, molecular function; HDL, high-density lipoproteins; IDL, intermediate-density lipoproteins; LDL, low-density lipoproteins; VLDL, very-low-density lipoproteins.

**Supplemental Figure 4**

**Figure S4.** **Propensity score matching and competing risk analysis of the UK Biobank data.**

**(A)** Stacked bar plots showing the number and composition of metabolites significantly associated with the risk of CAD and AAA after PSM. **(B)** Plot showing the associations of the routine lipid metabolites with the risk of CAD and AAA after PSM. **(C)** Sub-distribution hazard ratios (sHR) for routine lipid metabolites in the association with the risk of CAD and AAA, using Fine-Gray competing risk models. **(D)** Sensitivity analysis of heterogeneity in metabolite associations with incident CAD and AAA using a control-group sample-splitting approach. The control group was randomly split into two independent subsets for the Control *vs.* CAD and Control *vs.* AAA comparisons to avoid covariance arising from shared controls. Heterogeneity between β_CAD_ and β_AAA_ was evaluated using a Wald test. Hazard ratios per SD increment are shown with 95% confidence intervals as horizontal (AAA) and vertical (CAD) error bars. Metabolites with *P* ≤0.01 for heterogeneity between associations with CAD and AAA are marked by red dots. Abbreviations: AAA, abdominal aortic aneurysm; CAD, coronary artery disease; HR, hazard ratio; HDL, high-density lipoprotein; IDL, intermediate-density lipoproteins; LDL, low-density lipoproteins; VLDL, very-low-density lipoproteins.

**Supplemental Figure 5**

**Figure S5. Metabolomics reveals disease-specific metabolic responses in AS and AAA mice.**

**(A)** PCA score plots showing the metabolomics profiles of plasma samples from mice among control, AS and AAA groups (*n*=6 per group). **(B, C)** Volcano plots showing differential metabolites identified in plasma in AS and AAA mice compared to controls. Differential metabolites were identified using *P <* 0.05 and VIP > 1. **(D, E)** Pathway analysis of differentially expressed metabolites. Each bubble represents a metabolic pathway, with the size indicating the pathway impact and the color reflecting significance. Abbreviations: AAA, abdominal aortic aneurysm; AS, atherosclerosis; PCA, principal component analysis.

**Supplemental Figure 6**

**Figure S6. Differential transcriptomic and** **metabolomic regulation in HFD-induced AS and CaPO_4_-induced AAA.**

**(A)** Schematic illustration of the animal experimental design. **(B)** Representative images of macroscopic features and ORO staining of abdominal aortas. **(C)** Volcano plot showing the magnitude and significance of gene expression changes in the HFD-induced AS mice compared to controls (*n*= 3 mice in the HFD-induced AS group and *n*= 4 mice in the control group). DEGs were defined as *P <* 0.05 and FC ≥ 2. **(D, E)** Selected categories identified from **(D)** GO analysis and **(E)** Reactome analysis of upregulated and downregulated genes in the HFD-induced AS mice compared to controls. **(F)** Volcano plot showing the magnitude and significance of gene expression changes in the CaPO_4_-induced AAA mice compared to controls (*n*= 3 mice in CaPO_4_-induced AAA group and *n*= 4 mice in the control group). **(G, H)** Selected categories identified from **(G)** GO analysis and **(H)** Reactome analysis of upregulated and downregulated genes in the CaPO_4_-induced AAA mice compared to controls. DEGs were defined as *P <* 0.05 and FC ≥ 2. **(I)** PCA score plots showing metabolomic profiles of plasma samples from mice among control, AS and AAA groups (*n*=6 per group). **(J)** Pathway analysis of differentially expressed metabolites. Each bubble represents a metabolic pathway, with size indicating pathway impact and color representing significance. Abbreviations: AS, atherosclerosis; AAA, abdominal aortic aneurysm; DEGs, differentially expressed genes; FC, fold change; GO, gene ontology; PCA, principal component analysis.

**Supplemental Figure 7**

**Figure S7. A gene screening strategy based on GEO datasets.**

**(A)** Venn diagram showing the overlap of DEGs and 4 co-expressed genes identified from the GSE57691 microarray dataset and mouse RNA-seq data. **(B)** The mRNA expression of 4 candidate genes in abdominal aortas from WI-injury AS mice (*n*=6 per group). Data were analyzed by Student's *t*-test. **(C)** Upper panel: Quantification of *ACADM* mRNA and ACADM protein expression measured by RT-qPCR and Western blot in human AS and normal control segments (*n*=5 per group). Data were analyzed by Student's *t*-test. Lower panel: ACADM protein expression assessed by Western blot in human AS and normal control segments. **(D)** Upper panel: Quantification of *ACADM* mRNA and ACADM protein expression measured by RT-qPCR and Western blot in human AAA and normal control segments (*n*=5 per group). Data were analyzed by Student’s *t-*test. Lower panel: ACADM protein expression assessed by Western blot in human AAA and normal control segments. **(E)** Representative immunofluorescence images of ACADM expression in human AS versus normal control segments, co-stained with macrophage marker F4/80, smooth muscle cell marker αSMA, and DAPI. **(F)** Representative immunofluorescence images of ACADM expression in human AAA versus normal control segments, co-stained with macrophage marker F4/80, smooth muscle cell marker αSMA, and DAPI. **(G, H)** *Acadm* mRNA and ACADM protein expression measured by RT-qPCR and Western blot in isolated BMDMs stimulated with ox-LDL (100 µg/mL, 24 h; *n*=6 independent experiments). Data were analyzed using one-way ANOVA, followed by Tukey's *post-hoc* test. Abbreviations: DEG, differentially expressed gene; GEO, Gene Expression Omnibus; ox-LDL, oxidized low-density lipoprotein; BMDM, bone marrow-derived macrophage.

**Supplemental Figure 8**

**Figure S8.** **Medium-chain acylcarnitines are elevated in AS but not in AAA** **in both mouse and human samples.**

**(A)** Heatmaps showing the relative abundance of acylcarnitine species in mouse aortic tissues. **(B)** Quantification of representative medium-chain acylcarnitines (hexanoylcarnitine [C6], octanoylcarnitine [C8], and decanoylcarnitine [C10]) in mouse aortic tissues (*n*=5 per group). Data were analyzed using one-way ANOVA, followed by Tukey's *post-hoc* test. **(C)** Heatmaps showing the relative abundance of acylcarnitine species in mouse plasma. **(D)** Quantification of representative medium-chain acylcarnitines (hexanoylcarnitine [C6], octanoylcarnitine [C8], and decanoylcarnitine [C10]) in mouse plasma (*n*=5 per group). Data were analyzed using one-way ANOVA, followed by Tukey's *post-hoc* test. **(E)** Heatmaps showing the relative abundance of acylcarnitine species in human plasma. **(F)** Quantification of representative medium-chain acylcarnitines (hexanoylcarnitine [C6], octanoylcarnitine [C8], and decanoylcarnitine [C10]) in human plasma (*n*=5 per group). Data were analyzed using one-way ANOVA, followed by Tukey's *post-hoc* test. Abbreviations: AS, atherosclerosis; AAA, abdominal aortic aneurysm; CAD, coronary artery disease.

**Supplemental Figure 9**

**Figure S9.** **LD clearance by LD****∙ATTEC is autophagy-dependent.**

**(A)** **Top**: Representative images and quantification of LDs (stained with BODIPY 493/503; green), autophagosomes (LC3B; red), and nuclei (DAPI; blue) in BMDMs. LD∙ATTEC induced significant partial colocalization of LDs and autophagosomes. **Bottom**: Quantification of the percentage of LDs colocalized with autophagosomes (*n*=6 independent experiments). Data were analyzed using the Student's *t*-test. **(B)** Similar analysis to **(A)**, but performed on aortic segments from mice (AS_Vehicle and AS_LD∙ATTEC mice) 2 weeks after vehicle or LD∙ATTEC treatment (*n*=6 independent experiments). Data were analyzed using the Student's *t*-test. **(C)** Representative images and quantification of BODIPY493/503 staining of LDs in BMDMs treated with an autophagy inhibitor for 24h following 24h stimulation with ox-LDL (100 µg/mL). The LD number per cell and average LD size were determined (*n*=6 independent experiments). Data were analyzed using two-way ANOVA, followed by Tukey's *post-hoc* test. **(D)** Quantification of *Atg5* mRNA expression assessed by RT-qPCR in BMDMs after transfection with SiCon and Si*Atg5* (*n*=6 independent experiments). Data were analyzed by Student's *t*-test. **(E)** Representative images and quantification of BODIPY493/503 staining of the LDs in the WT versus Atg5-knockout BMDMs treated with LD∙ATTEC for 24h following 24h stimulation with ox-LDL (100 µg/mL). The LD number per cell and average LD size were analyzed (*n*=6 independent experiments). Data were analyzed using two-way ANOVA, followed by Tukey's *post-hoc* test. Abbreviations: BMDM, bone marrow-derived macrophage; LD, lipid droplet.

**Supplemental Figure 10**

**Figure S10. Lipid droplet deposition, blood pressure and plasma lipids in AS and AAA mice treated with vehicle or LD∙ATTEC.**

**(A) Left,** Representative photomicrographs of abdominal aortic frozen crossed-sections from wire injury-induced AS mice and the Ang II-induced AAA mice stained with ORO**. Right,** Quantification of the lipid deposition (percentage of lesion area; *n*=6). Data were analyzed by one-way ANOVA, followed by Tukey's *post-hoc* test. **(B)** Systolic blood pressure measurements at 0, 7, 14, 21, and 28 days following treatment with either vehicle or LD∙ATTEC in AS and AAA mice (*n*=10 per group). Group differences over time were analyzed using generalized estimating equations (GEE). **(C-E)** Plasma levels of TG, TC, and LDL‐C in AS and AAA mice after 4 weeks of treatment with vehicle or LD∙ATTEC (*n*=10 per group). Data were analyzed by two-way ANOVA followed by Tukey's multiple-comparison test. Abbreviations: AS, atherosclerosis; AAA, abdominal aortic aneurysm; TG, triglycerides; TC, total cholesterol; LDL-C, low-density lipoprotein-cholesterol; ORO, Oil Red O.

**Supplemental Figure 11**

**Figure S11. LD∙ATTEC treatment reduces lipid droplets but fails to rescue AAA-relevant phenotypes in mice.**

**(A)** Representative images of abdominal aortas visualized by MUI using the B mode and histological staining (ORO, HE, and Masson) of cross-sections of aneurysms. **(B)** Quantification of the maximal diameter of abdominal aortas measured by MUI using the B mode in the indicated groups (*n*=14 per group). **(C, D)** Quantification of collagen contents (percentage of lesion area, *n*=7 per group) **(D)** and lipid deposition (percentage of lesion area, *n*=10 per group) **(D)**. Data were analyzed by Mann-Whitney *U* test (**B**) or Student's *t*-test (**C** and **D**). Abbreviations: Ang II, angiotensin II; AAA, abdominal aortic aneurysm; AS, atherosclerosis; HE, hematoxylin and eosin; Masson, Masson’s trichrome; MUI, micro-ultrasound imaging.

**Supplemental Figure 12**

**Figure S12. LD∙ATTEC treatment leads to different metabolomic changes between AS and AAA mouse models.**

**(A)** PCA score plots of the metabolomic profiles of plasma samples from mice in the indicated groups (*n*=8 mice per group for AAA_Vehicle and AAA_LD∙ATTEC; *n*=7 mice per group for AS_Vehicle and AS_LD∙ATTEC). **(B)** Proportions of differential metabolites. Left panel: AS_Vehicle group versus AS_LD∙ATTEC group. Right panel: AAA_Vehicle group versus AAA_LD∙ATTEC group. **(C)** Volcano plot showing differential metabolites between groups. **(D)** Heatmap of top 20 differential metabolites in the indicated groups. **(E)** Quantification of the acylcarnitine levels (e.g., L-Carnitine, O-Acetylcarnitine, Dodecanoylcarnitine, Propionylcarnitine, cis-5-Tetradecenoylcarnitine) in the plasma of the indicated groups. Data were analyzed by Student's *t*-test (normally distributed variables) or Mann-Whitney *U* test (non-normally distributed variables). Abbreviations: AS, atherosclerosis; AAA, abdominal aortic aneurysm; PCA, principal component analysis.

**Supplemental Figure 13**

**Figure S13. LD∙ATTEC treatment markedly reduces lipid contents in AS mice.**

**(A)** PCA of the metabolomic profiles between the AS_Vehicle and AS_LD∙ATTEC groups (*n*=5 mice per group). **(B)** Volcano plots showing differential lipid metabolites between the AS_Vehicle and AS_LD∙ATTEC groups. **(C)** Pie charts showing the composition of the altered lipid metabolites by lipid classes between the AS_Vehicle and AS_LD∙ATTEC groups. **(D)** Bar plots showing the degree of lipid metabolite changes between the AS_Vehicle and AS_LD∙ATTEC groups. The red bar represents the most significantly decreased lipid subclasses in the AS_LD∙ATTEC compared to the AS_Vehicle group. **(E)** Dot plots showing log_2_ fold changes in lipid subclasses between the AS_Vehicle and AS_LD∙ATTEC groups, and the corresponding *P* values displayed as −log_10_ (*P*-value). Dot colors correspond to different lipid subclasses, and the dot size indicates significance. Each dot represents a lipid metabolite. Only lipids with *P <* 0.05 are shown. **(F–I)** Quantification of the TAG, DAG, CE, and FFA levels in the aortas from mice in the indicated groups. Data were analyzed by Student's *t*-test (for normally distributed variables) or Mann-Whitney *U* test (for non-normally distributed variables). Abbreviations: CE, cholesterol esters; CAD, coronary artery disease; DAG, diglycerides; FFA, free fatty acids; HCER, hexosylceramides; LPC, lysophosphatidylcholines; LPE, lysophosphatidylethanolamines; PC, phosphatidylcholines; PE, phosphatidylethanolamines; PG, phosphatidylglycerol; PI, phosphatidylinositol; TAG, triglycerides; PCA, principal component analysis.

**Supplemental Figure 14**

**Figure S14.** **Generation of myeloid cell–specific *Acadm* overexpression mice.** Schematic diagram of the construction of conditional myeloid cell-specific *Acadm* overexpression mice (*Acadm^flox/flox^Lysm-Cre*, *Acadm^Tg^*).

**Supplemental Figure 15**

**Figure S15. Association between *ACADM* loss-of-function mutations and carotid plaque burden** **in the UK Biobank cohort.**

**(A)** Schematic workflow for testing the association of *ACADM* predicted loss-of-function (pLoF) variants with carotid plaque burden in the UK Biobank. pLoF variants in *ACADM* were identified from UK Biobank whole-exome sequencing data. Participants carrying *ACADM* pLoF variants were compared with noncarriers in association analyses to assess their relationship with plaque burden, measured by carotid intima–media thickness (cIMT). **(B)** Comparison of cIMT between carriers and noncarriers of pLoF variants in *ACADM*. Data were analyzed using linear regression adjusted for age, sex, assessment center, principal components (PC1–PC10), and genotype array. **(C)** Effect estimates (β) with 95% confidence intervals (CI) and corresponding *P*-values. Abbreviations: cIMT, carotid intima–media thickness; CI, confidence interval; pLoF, predicted loss of function.

**Supplemental Figure 16**

**Figure S16.** ***Acadm* overexpression preserves mitochondrial homeostasis** **in BMDMs under ox-LDL stimulation**.

**(A)** Intracellular acetyl-CoA, free-CoA, and free fatty acid levels in BMDMs with or without *Acadm* overexpression (*n*=6 independent experiments). Data were analyzed by two-way ANOVA followed by Tukey's *post-hoc* test. **(B)** Summarized OCR tracings in BMDMs transduced with *Acadm*-overexpression or control lentivirus, followed by ox-LDL stimulation (100ug/ml, 24h) (*n*=6 independent experiments). **(C-E)** Quantification of basal, maximal and ATP-coupled OCRs in indicated groups (*n*=6 independent experiments). Data were analyzed by two-way ANOVA followed by Tukey's *post-hoc* test. **(F, G)** Representative images and quantitative analysis of mitochondrial membrane potential assessed by Mito Tracker Green staining in PBS- or ox-LDL–treated BMDMs with or without *Acadm* overexpression (*n*=5 independent experiments). Data were analyzed by two-way ANOVA followed by Tukey's *post-hoc* test. **(H, I)** Representative images and quantitative analysis of mitochondrial ROS levels assessed by MitoSOX staining in PBS- or ox-LDL–treated BMDMs with or without *Acadm* overexpression (*n*=5 independent experiments). Data were analyzed by two-way ANOVA followed by Tukey's *post-hoc* test. Abbreviations: ATP, adenosine triphosphate; BMDM, bone marrow-derived macrophage; ox-LDL, oxidized low-density lipoprotein; OCR, oxygen consumption rate; ROS, reactive oxygen species.

**Supplemental Figure 17**

**Figure S17. Machine learning model utilizing lipidomic features for the diagnosis of CAD.**

**(A)** OR values of predictor variables and their 95% confidence intervals in the logistic model incorporating lipidomic features for CAD *vs.* HC in the training cohort (*n*=169). **(B)** Nomograms constructed based on the training cohort for CAD *vs.* HC. **(C)** Calibration curves of the logistic regression analysis in the training cohort for CAD *vs.* HC. Calibration intercept = 0.001 (SE 0.306, *P* > 0.99); calibration slope = 1.006 (SE 0.182, *P* = 0.974). Joint test of intercept = 0 and slope = 1: χ² = 0.0001 (df = 2, *P* > 0.99). No statistically significant deviation from ideal calibration was observed. **(D)** ROC curve for four predictive models in the validation cohort (*n*=73) for CAD *vs.* HC. **(E)** Radar plot of evaluation metrics for machine learning diagnostic models for CAD *vs.* HC. **(F)** Boxplots of concentration of the identified key lipid metabolites with significant coefficients in the logistic regression analysis. Data were analyzed by Student's *t*-test. Abbreviations: AUC, area under receiver operating characteristic curve; CAD, coronary artery disease; CE, cholesterol esters; TAG, triglycerides; HC, healthy control; OR, odds ratio; ROC, receiver operating characteristic curve; SVM, support vector machine; XGBoost, eXtreme Gradient Boosting.

**Supplemental Figure 18**

**Figure S18. Machine learning model utilizing lipidomic features for the diagnosis of AAA.**

**(A)** OR values of predictor variables and their 95% confidence intervals in the logistic model incorporating lipidomic features for AAA *vs.* HC in the training cohort (*n*=169). **(B)** Nomograms constructed based on the training cohort for AAA *vs.* HC. **(C)** Calibration curves of the logistic regression analysis in the training cohort for AAA *vs.* HC. Calibration intercept < 0.001 (SE 0. 225, *P* > 0.99); calibration slope = 1.001 (SE 0.157, *P =* 0.995). Joint test of intercept = 0 and slope = 1: χ² < 0.001 (df = 2, *P* > 0.99). No statistically significant deviation from ideal calibration was observed. **(D)** ROC curve for four predictive models in the validation cohort (*n*=73) for AAA *vs.* HC. **(E)** Radar plot of evaluation metrics for machine learning diagnostic models for AAA *vs.* HC. **(F)** Boxplots of concentration of the identified key lipid metabolites with significant coefficients in the logistic regression analysis. Data were analyzed by Student's *t*-test. Abbreviations: AAA, abdominal aortic aneurysm; AUC: area under receiver operating characteristic curve; HC, healthy control; SVM: support vector machine; XGBoost: eXtreme Gradient Boosting; OR, odds ratio. FFA, free fatty acids; HCER, hexosylceramides; LPC, lysophosphatidylcholines; LPE, lysophosphatidylethanolamines; PC, phosphatidylcholines; PE, phosphatidylethanolamines; SM, sphingomyelins.

**Supplemental Figure 19**

**Figure S19.** **Overview of the experimental workflow.**
